# *De novo* genome assembly of *Solanum sitiens* reveals structural variation associated with drought and salinity tolerance

**DOI:** 10.1093/bioinformatics/btab048

**Published:** 2021-01-30

**Authors:** Corentin Molitor, Tomasz J Kurowski, Pedro M Fidalgo de Almeida, Pramod Eerolla, Daniel J Spindlow, Sarvesh P Kashyap, Bijendra Singh, H C Prasanna, Andrew J Thompson, Fady R Mohareb

**Affiliations:** The Bioinformatics Group, School of Water, Energy and Environment, Cranfield University, Bedford MK43 0AL, UK; The Bioinformatics Group, School of Water, Energy and Environment, Cranfield University, Bedford MK43 0AL, UK; The Bioinformatics Group, School of Water, Energy and Environment, Cranfield University, Bedford MK43 0AL, UK; The Bioinformatics Group, School of Water, Energy and Environment, Cranfield University, Bedford MK43 0AL, UK; The Bioinformatics Group, School of Water, Energy and Environment, Cranfield University, Bedford MK43 0AL, UK; Division of Crop Improvement, ICAR-Indian Institute of Vegetable Research, Varanasi, India; Division of Crop Improvement, ICAR-Indian Institute of Vegetable Research, Varanasi, India; Division of Crop Improvement, ICAR-Indian Institute of Vegetable Research, Varanasi, India; Division of Vegetable Crops, ICAR-Indian Institute of Horticultural Research, Bangalore, India; The Bioinformatics Group, School of Water, Energy and Environment, Cranfield University, Bedford MK43 0AL, UK; The Bioinformatics Group, School of Water, Energy and Environment, Cranfield University, Bedford MK43 0AL, UK

## Abstract

**Motivation:**

*Solanum sitiens* is a self-incompatible wild relative of tomato, characterized by salt and drought-resistance traits, with the potential to contribute through breeding programmes to crop improvement in cultivated tomato. This species has a distinct morphology, classification and ecotype compared to other stress resistant wild tomato relatives such as *S.pennellii* and *S.chilense*. Therefore, the availability of a reference genome for *S.sitiens* will facilitate the genetic and molecular understanding of salt and drought resistance.

**Results:**

A high-quality *de novo* genome and transcriptome assembly for *S.sitiens* (Accession LA1974) has been developed. A hybrid assembly strategy was followed using Illumina short reads (∼159× coverage) and PacBio long reads (∼44× coverage), generating a total of ∼262 Gbp of DNA sequence. A reference genome of 1245 Mbp, arranged in 1483 scaffolds with an N50 of 1.826 Mbp was generated. Genome completeness was estimated at 95% using the Benchmarking Universal Single-Copy Orthologs (BUSCO) and the K-mer Analysis Tool (KAT). In addition, ∼63 Gbp of RNA-Seq were generated to support the prediction of 31 164 genes from the assembly, and to perform a *de novo* transcriptome. Lastly, we identified three large inversions compared to *S.lycopersicum*, containing several drought-resistance-related genes, such as *beta-amylase 1* and *YUCCA7.*

**Availability and implementation:**

*S.sitiens* (LA1974) raw sequencing, transcriptome and genome assembly have been deposited at the NCBI’s Sequence Read Archive, under the BioProject number ‘PRJNA633104’. All the commands and scripts necessary to generate the assembly are available at the following github repository: https://github.com/MCorentin/Solanum_sitiens_assembly.

**Supplementary information:**

Supplementary data are available at *Bioinformatics* online.

## 1 Introduction

Cultivated tomato (*Solanum lycopersicum* L.) is grown commercially as an irrigated field crop for processing and fresh fruit production. Relative to many crops it is sensitive to soil water deficits, especially at the flowering stage (Atherton and Rudich, 1986), and therefore pressure on water resources (FAO, 2013) makes high yield under water deficit conditions, and high water use efficiency (yield per unit water input) important breeding targets (Robledo *et al.*, 2020). The physiological traits required to achieve these broad targets are complex (Tardieu *et al.*, 2018) and require deep understanding of water flows within the soil-plant-atmosphere system and phenology (relationship between crop development and climate); smart breeding requires a knowledge of the genetic loci and their interactions to control specific contributing plant traits (Zsogon *et al.*, 2017, 2018).

Crop wild relatives are an important source of genetic variation for crop improvement; they have been deployed successfully to improve e.g. disease resistance, flavour and fruit size in cultivated tomato in recent decades (Schouten et al., 2019); there remains the opportunity to transfer adaptations to abiotic stresses such as drought and salinity from wild species to tomato crops. Tomato and its closest wild relatives are classified into *Solanum* section *Lycopersicon* containing 13 species (Peralta et al., 2008), including three species with drought-resistant accessions: *S.pennellii*, *S.pimpinellifolium* and *S.chilense* (Zsögön et al., 2017). There is a fourth drought-resistant species, *S.sitiens* (Chetelat *et al.*, 2009), but this is considered to be within an outgroup, *Solanum* sect. *Lycopersicoides*. The latter contains only one other species, *S.lycopersicoides* (Peralta *et al.*, 2008), and both members of this section can be hybridized with cultivated tomato by overcoming significant reproductive barriers (Chetelat, 2016; DeVerna *et al.*, 1990; Pertuze *et al.*, 2003).

Of the drought-resistant species, excellent genetic and genomic resources (including *de novo* assembly) exist for *S.pennellii* (Bolger *et al.*, 2014; Schmidt *et al.*, 2017) where highly successful strategies have been employed to exploit allelic variation (Alseekh *et al.*, 2013), and assemblies have been recently reported for *S.chilense* LA3111 (Stam *et al.*, 2019) and *S.pimpinellifolium* LA0480 (Razali *et al.*, 2018). Extensive resequencing data mapped against cultivated tomato is available from over 500 accessions, including all wild species (Kevei *et al.*, 2015; Lin *et al.*, 2014; Tomato Genome Consortium, 2012), and a pan-genome has been created for tomato using 725 accessions (Gao *et al.*, 2019), but this included limited numbers of wild species and only within the *Solanum* sect. *Lycopersicon*. A genome assembly for *S.sitiens* is lacking, therefore, the aim of this work was to create this genomic resource to underpin use of this species in genetic studies of drought and salinity resistance.

*Solanum sitiens* is found in an extremely dry habitat of Chile, within a very limited geographic range on the plateau of the hyper arid Atacama Desert, with most accessions collected between 2500 and 3300 m in altitude (Chetelat *et al.*, 2009). The C.M. Rick Tomato Genetic Resources Centre (University of California at Davis), indicates resistance to drought stress as a general feature of *S.sitiens*, and recommends accessions LA1974 and LA2876 for investigations related to drought. Accession LA1974, the object of this study, was collected by Carlos Ochoa from Chuquicamata, Antofagasta, Chile in 1979 from an extremely dry habitat (Pertuze *et al.*, 2002). The collection sites recorded for *S.sitiens* are typically arid and with soil salinity at levels where cultivated tomatoes would not be productive (Chetelat *et al.*, 2009). Descriptions of the morphological adaptations of *S.sitiens* to drought are reported: thick leathery leaves that are small and able to fold along the mid-vein, and the ability to regenerate from the base of the stem after prolonged drought. However, there are no known studies of drought physiology in this species. In addition, *S.sitiens* has a seed dispersal strategy unique within the *Solanum* genus in which fruits desiccate, rather than soften and ripen, and then drop to the ground for dispersal by wind or by rolling down slopes (Chetelat *et al.*, 2009). Recently, a library of 56 introgression lines representing 93% of the genome of *S.sitiens* accessions LA4331 and LA1974 was reported (Chetelat *et al.*, 2009, 2019). The availability of a reference genome assembly, in addition to these new genetic resources in *S.sitiens*, will benefit breeding efforts in cultivated tomato and open new opportunities for further studies linking genes to the unique biology of this species.

*Solanum sitiens* is allogamous and self-incompatible, so likely to be highly heterozygous, making genome assembly more challenging than for inbred species. A hybrid assembly strategy, involving both short and long reads as well as optical mapping, was applied here. The advantage of such an approach is to combine the low error rates of short reads with the structural information contained within the long reads and optical mapping, thus resulting in a more precise and less fragmented assembly (Powell *et al.*, 2020; Scott *et al.*, 2020).

## 2 Materials and methods

See Supplementary Materials S1.

## 3 Results

### Data quality control

Supplementary Table S5 describes the length distribution of the filtered BioNano molecules and the number of labels/100 kbp, which are in the optimal range recommended by BioNano, between 8 and 15 (Bionano Prep^TM^Labeling-NLRSProtocol).

### The assembly

The statistics obtained at each step of the pipeline are available in Table 1. More details are available in Supplementary Table S7.

**Table 1.** Statistics of the assembly at different stages

Stage	Length (Mbp)	# contigs/scaffolds	Largest scaffold (bp)	N50 (bp)
MaSuRCA	1255	5492	11 182 222	805 210
SSPACE	1262	3516	11 182 222	979 616
Hybrid Scaffold	1275	3355	14 576 794	1 183 603
Pilon	1274	3355	14 564 673	1 183 602
GapFiller	1274	3355	14 565 231	1 184 430
Dedupe	1245	2153	14 565 231	1 214 320
**Arcs + Links**	**1245**	**1481**	**22 444 909**	**1 826** **367**

*Note*: Results obtained with Quast v4.5. Values are expressed in Mbp. The final assembly statistics are in bold.

Pilon corrected the assembly based on the evidence from the alignments with the Illumina reads, for both libraries >98% of the reads were successfully mapped. Pilon corrected 127 915 single bases, 24 066 insertions amounting to 163 484 bp and 28 397 deletions amounting to 437 669 bp.

GapFiller managed to remove ∼400 000 N’s from the assembly by resizing gaps. There were 18 797 250 N’s remaining after the GapFiller step, corresponding to ∼1.5% of the assembly length.

The blast search of our scaffolds against NCBI-nr matched *Solanum* accessions only. Most of the hits correspond to *S.pennellii*, and nine scaffolds had no hits, interestingly, these were removed during the deduplication step (see below). The e-value distribution of the hits is available as Supplementary Figure SF3A.

As shown by Busco, some artificial duplications were present in the assembly, notably due to the high heterozygosity rate. BBmap dedupe.sh removed 1202 duplicated scaffolds from the assembly, amounting to ∼29 Mbp. Among these, all the scaffolds without blast hits found previously were removed as duplication during this step. Moreover, most of the scaffolds with a percentage of confirmed bases lower than 95%, as detected by Pilon, were removed (see Supplementary Fig. SF4) and the average percentage of confirmed bases, increased from 95% to 98%.

### The final assembly quality assessment

The final assembly is composed of 1483 scaffolds for a total length of 1245 Mbp, close to the previously estimated genome size of 1129 Mbp. There are 17 566 158 unknown bases (1.4% of the total length) and 35.34% of the bases are Guanine or Cytosine (GC). The N50 of 1 826 367 bp was found at the 186^th^ scaffold, with the largest sequence being 22 444 909 bp long.

From the 3052 genes in BUSCO’s Solanaceae dataset, 2898 (95%) were found in the assembly as complete, this BUSCO result parallels with KAT estimated assembly completeness of 97%, as described below.

KAT estimated a genome size of 841 Mbp, we suspect an underestimation of the size due to KAT ignoring k-mers found outside the heterozygous and homozygous peaks, however, this does not affect the estimation of assembly completeness of 97.28%, which is based on the missing k-mers in the homozygous peak. From the Illumina reads, KAT found three peaks at multiplicity 48, 97 and 192, the first two peaks correspond to the heterozygous and homozygous peaks respectively, and the last peak indicates the presence of regions present in two copies in *S.sitiens* (see Supplementary Fig. SF2). The details of KAT output are available in Supplementary Table ST1.

The overall mapping rates of the 4 RNA-Seq libraries against the assembly were between 91.91% and 94.86%. Similar to the approach taken by Bolger *et al.* (2014) the unmapped reads were re-mapped to *S.lycopersicum* v3.0 to determine if they were due to assembly incompleteness, only 1.32–1.76% of the reads mapped to *S.lycopersicum* and not our assembly, indicating, therefore, no loss of data due to assembly incompleteness.

### 3.1 Comparison with closely related assemblies

The genome assembly overall structure and completeness was assessed further by performing an overall pairwise alignment against *S.lycopersicum* and *S.pennelliii*. Supplementary Figure SF3 shows the alignment of our *S.sitiens* scaffolds against all twelve chromosomes of *S.lycopersicum*. It can be noted that the developed *S.sitiens* assembly is showing an overall continuity across all chromosomes*.* Similar pairwise alignment against *S.pennellii* is also available as Supplementary Figure SF5 and similar coverage was also obtained.

Statistics of our MaSuRCA assembly were compared against the contig assemblies of *S.pennellii*, *S.pimpinellifolium* and *S.lycospersicum* v3.0, see Table 2. There were obtained with Quast using the ‘–scaffold’ option to break the scaffolds by stretch of Ns longer than 10. This allowed fair comparison between the assemblies at the scaffold and chromosome level. A BUSCO analysis on the original assembly fasta files, allowed comparison between our assembly and the assemblies of *S.lycopersicum* v3.0, *S.pennelllii, S.pimpinellifolium*. See Supplementary Table ST2 for more details on the Busco results.

**Table 2.** Comparison at the contig level of our *S.sitiens* assembly against *S.pennellii* and *S.lycopersicum*

Assembly	# contigs	Largest contig (kbp)	N50 (kbp)	# N's per 100 kbp	% complete (Busco*)
*S.sitiens*	4002	14 565	844.4	0.03	95.0%
*S.lycopersicum v3.0*	22 691	3167	133.0	0.01	96.7%
*S.pennellii*	33 537	491.7	58.7	1.53	96.9%
*S.pimpinellifolium*	133 578	347	25.6	2.87	94.3%

*Note*: The scaffolds were broken at every 10 continuous N's. *The Busco analysis was performed on the original fasta files.

### *De novo* transcriptome assembly and functional annotation

Quality assessment for raw reads was performed using Rcorrector. 8.4% of the RNA-seq reads were identified as ‘unfixable’; and were marked as such should the number of detected errors across the length of the read exceed a pre-determined threshold. These unfixable reads may either be derived from a lowly expressed transcript of which a more greatly expressed homolog is present, or may simply contain too many errors to be corrected. To avoid the inclusion of damaging k-mers, all unfixable reads were removed. The error correction of the reads with low quality did not remove any additional reads, but trimmed 1.62% of the base pairs by cutting off the adapters. Ultimately, the cleaned data retained in average 32 million PE reads per sample.

The raw transcriptome from Trinity generated 461 083 contigs for a total size of 387 Mbp and an N50 of 1710 bp. This is obviously much higher than the expected number of transcripts and is usually the case with unfiltered output from Trinity. Moreover, high levels of duplication are common and to be expected in transcriptome assembly due to the transcription of multiple isoforms of the same RNA-product. Therefore, duplicates and lowly expressed transcripts were considered as artefacts and were subsequently removed. CD-HIT clustered similar sequences and removed 118 638 duplicates. The transcriptome was filtered by a threshold of 1.5 TPM, which was the optimum compromise between duplication level, transcript count and completeness. The final transcriptome contains 131 581 transcripts, with an estimated BUSCO completeness of 90.0%. More details about the *de novo* transcriptome assembly statistics are available in Supplementary Table ST3.

The functional annotation was done by aligning the transcriptome against NCBI-nr and custom blast databases produced from the assemblies of closely related species. During the InterProScan search, 77 834 transcripts had blast hits, including 35 645 transcripts associated with GO terms. InterproScan repeats distribution results showed Leucine-rich repeat (LRR) proteins repeating 456 times. This domain is directly involved in recognizing the presence of pathogen-associated molecular patterns (PAMPs). The pathogen products of avirulence (AVR) genes are recognized by the nucleotide-binding site of LRR proteins. A gene coding the protein with Ankyrin repeats domain found 169 times in InterproScan results shows increased resistance to disease and spontaneous cell-death. Spontaneous cell death is a critical disease response mechanism where a plant kills the diseased cells instantly to block the disease from spreading. An in-depth look at how and when these genes are over-expressing in *S.sitiens* can provide more understanding of its response to pathogens and diseases.

### Gene prediction from the genome assembly

Excluding gaps from the assembly, RepeatMasker masked 70% of the genome as repetitive elements, which is in the range of other *Solanum* accessions, e.g. 59.5% for *S.pimpinellifolium* (Razali *et al.*, 2018), 64% for *S.lycopersicum* v4 (Hosmani *et al.*, 2019) and 82% for *S.pennellii* (Bolger *et al.*, 2014).

Augustus predicted 31 164 genes, corresponding to 33 386 transcripts, using hints about the intron positions obtained from the alignment of the RNA-seq against our genome assembly. This number is very close to the number of coding genes present in *S.lycopersicum* (34 658) and *S.pennellii* (32 273).

### Comparative orthology

Orthofinder revealed 23 225 orthogroups in total between *S.sitiens, S.chilense, S.pennnellii, S.pimpinellifolium, S.lycopersicum, S.tuberosum* and *A.thaliana*, defined as the set of genes descended from a singular gene of the last common ancestor of each species. A rooted phylogenetic tree was inferred through the presence of the most closely related genes within single and multi-copy orthogroups (see Supplementary Fig. SF4B). As expected *S.pimpinellifolium* and *S.lycopersicum*, which present only 0.6% nucleotide divergence and recent introgressions (Gao *et al.*, 2019), are shown to be the most closely related of the tomato species whereas the potato, *S.tuberosum*, is the least closely related species to the domestic tomato. The correct placement of these three species lends credence to the accuracy of the placement of *S.sitiens* and *S.chilense* within the phylogram and the output of Orthofinder. Of the five tomato species considered, 13 536 orthogroups were common to all species (see Supplementary Fig. SF4A). A significant number of orthogroups were found to be unique to each species, evidence of the genetic diversity between tomatoes. However, it should be noted that individual tomatoes will harbour different genes as revealed through the investigation of the tomato pan-genome (Gao *et al.*, 2019) and thus the extent of unique orthogroup assignment is to a certain extent dependent on the individuals from which the protein sequences are derived.

Some of the 561 transcripts belonging to the 320 orthogroups unique to *S.sitiens* (see Supplementary Fig. SF4A) have annotations related to salt and drought stress tolerance. Notably, *Glycolate Oxidase* (*GLO1* and *GLO4*), which are involved in drought stress response in *Vigna* and pea (Mittler and Zilinskas, 1994). One of the transcript mapped with the GO term ‘response to water deprivation’ (GO: 0009414) and blasted against an aquaporin *PIP1-2* which plays a role in drought tolerance (Shekoofa and Sinclair, 2018). Another transcript mapped to the GO term ‘response to water’ (GO: 0009415) and blasted against ‘abscisic acid and environmental stress-inducible protein *TAS14*’ which was found to improve both salt and drought resistance (Munoz-Mayor *et al.*, 2012). Other interesting transcripts blasted against *Oxophytodienoate Reductase 1* and *FQR1-like NAD(P)H dehydrogenase* which were shown to confer salt tolerance in wheat (Dong *et al.*, 2013) and Arabidopsis (Song *et al.*, 2016) respectively. Very interestingly, genes with high homology to *FQR1-like NAD(P)H dehydrogenase* were also identified in *S.pimpinellifolium*, another drought and salt tolerant tomato wild relative (Razali *et al.*, 2018). Further study of these unique orthologues might give insight into the adaptation of *S. sitiens* in water limited environments.

### 3.2 Identification of inversions against *S.lycopersicum*

Three potentially interesting inversions against *S.lycopersicum* were identified using the alignments produced with Mummer (see Supplementary Fig. S6). One is on scaffold95 against chromosome 11 of *S.lycopersicum* and is present both in our assembly and *S.pennellii.* This inversion has also been previously reported in *S.habrochaites* against *S.lycopersicum* (Wolters *et al.*, 2015). The other two, on scaffold11 and scaffold8, are unique to *S.sitiens* against both *S.lycopersicum* and *S.pennellii*. All three inversions were located within contiguous sequences on our scaffolds. The locations of these inversions were intersected with the gene prediction from Augustus. The genes found in the inversions were blasted against ITAG4.0 and TAIR10 and the top hits were searched in the literature for links to drought or salt resistance (See Supplementary Table ST4). The Arabidopsis orthologues obtained from the literature search were then queried against the ePlant web repository (Waese *et al.*, 2017) to look for expression changes during drought and salt stresses (‘Abiotic Stress eFP’ and ‘Abiotic Stress II eFP’ views under ‘Tissue & Experiment eFP viewers’) (Kilian *et al.*, 2007; Wilkins *et al.*, 2010).

As mentioned in the introduction, *S.sitiens* possess a unique fruit maturation process. Instead of ripening, the fruit are desiccating allowing the seeds to disperse in the desert. Interestingly, one of the genes in the inversion on scaffold11, *ARR9*, is involved in transcription of ABA biosynthetic genes, which in turn affects seed desiccation tolerance (Wohlbach *et al.*, 2008).

The most promising inversion is the one between scaffold8 and chromosome 9 of *S.lycopersicum*. It contains two genes annotated with the GO Term: ‘response to water deprivation’, *YUCCA7* and *BAM1*, making it a locus of interest for drought tolerance.

Some Arabidopsis orthologues of the genes identified in Supplementary Table ST4 change their expression during drought and salt stresses, as shown on ePlant. Notably, *allene oxide synthase* and *BAM1* are up-regulated in both drought and salt stresses. *YUCCA7* is following the same trend except in roots, where it is down regulated during salt stress. *ABC transporter* is up-regulated during salt stress. On the contrary, protein *disulfide-isomerase 5-1* and *ARR9* are down regulated during salt stress. *High-affinity nitrate transporter 2.2* seems to undergo a lot of change in expression during both drought and salt stresses. The ePlant figures are available as Supplementary Figure SF8.

We hypothesize that these inversions might affect the expression of the genes described in Supplementary Table ST4 and renders the plant more susceptible to drought and salt stresses. Further research will be needed to confirm and understand the interplay between these inversions and *S.sitiens* adaptation to its environment.

### 3.3 Pseudomolecule assemblies based on similar species

The *chromosome_scaffolder.sh* script from MaSuRCA, produced two *S.sitiens* chromosome scale assemblies, one based on *S.lycopersicum*, the other on *S.pennellii*. The assemblies’ statistics and quality were assessed with the same tools and parameters as the scaffold assembly and the results are available in Table 3. 81% and 83% of the total length was covered across 12 chromosome sequences for the assemblies based on *S.lycopersicum* and *S.pennellii* respectively. Moreover, the pseudomolecules assemblies were aligned with mummer against their respective references, with the same parameters as described in the methods section “Assessnebr of the assemvly quality” of the Supplementary Materials. The dotplots generated from the alignment are available as Supplementary Figures SF7A and B.

**Table 3.** Pseudomolecule assembly statistics

Assembly name	*Scaffold assembly*	*Si_SL3.0*	*Si_SP*
Length (Mbp)	1245	1703	1822
N50 (Mbp)	1.82	113.28	123.13
Largest scaffold (Mbp)	22.44	174.95	186.56
# scaffolds	1483	3042	2877
N/100 kbp	1411	27 964	32 724
KAT estimated completeness	97.28%	97.25%	97.22%
BUSCO completeness	94.9%	94.9%	94.9%

*Note*: Si_SL3.0: Pseudomolecule assembly using scaffolds anchored on *S.lycopersicum* 3.0 backbone. Si_SP: Pseudomolecule assembly using scaffolds anchored on *S.pennellii*.

While these pseudomolecules assemblies are not as accurate as if they were constructed purely from *S.sitiens* data, due to some potential misassemblies stemming from the differences between the genomes of *S.sitiens* and the two relative species used as references. Nevertheless, these assemblies can be beneficial for genotyping and visualization purposes via genome browsers.

## 4 Conclusions

Here, we present high-quality *de novo* genome and transcriptome assemblies of *S.sitiens*, a tomato wild relative. This scaffold assembly is more than 95% complete, as measured by BUSCO and KAT. Comparison at the contig level shows better contiguity than assemblies of similar Solanaceae species, which will facilitate consequent analyses, notably gene prediction, detection of structural variations and pseudomolecule assembly. Analysis of *S.sitiens* unique orthologues and three inversions against *S.lycopersicum* highlighted genes that could be involved in drought and salt tolerance, this will lead the way for future discoveries on the importance of these genes. Moreover, the availability of this reference is potentially helpful to assist the breeding efforts to integrate drought resistance in tomato crops.

## Supplementary Material

btab048_Supplementary_Data
