# Supplementary material for: De novo genome assembly of Solanum sitiens reveals structural variation associated with drought and salinity tolerance
Source: Bioinformatics. 2021 Jan 30;37(14):1941–5. doi: 10.1093/bioinformatics/btab048 (PMC8496510; doi:10.1093/bioinformatics/btab048)
Supplement: btab048_Supplementary_Data [file btab048_supplementary_data.docx]

*De novo* genome assembly of *Solanum sitiens reveals structural variation associated with drought and salinity tolerance*

Corentin Molitor^1^, Tomasz J. Kurowski^1^, Pedro M. Fidalgo de Almeida^1^, Pramod Eerolla^1^, Daniel J. Spindlow^1^, Sarvesh P. Kashyap^2^, Bijendra Singh^2^, HC Prasanna^2,3^, Andrew J. Thompson^1^, Fady R. Mohareb^1*^

^1^ The Bioinformatics Group, School of Water, Energy and Environment, Cranfield University, College Road, Bedford, MK43 0AL, UK.

^2^ Division of Crop Improvement, ICAR-Indian Institute of Vegetable Research, Varanasi, India.

^3^ Division of Vegetable Crops, ICAR-Indian Institute of Horticultural Research, Bangalore, India.

*To whom correspondence should be addressed.

**Supplementary Materials – S1: Materials and methods**

**Plant material**

Seeds of *S. sitiens* accession LA1974 were obtained from the C.M. Rick Tomato Genetics Resource Center maintained by the Department of Plant Sciences, University of California, Davis, USA. Seeds were treated with 50% v/v household bleach, equivalent to 2.25% w/v sodium hypochlorite for 60 mins, rinsed in tap water and then germinated on filter paper soaked in distilled water at 25^o^C in the dark. A single plant was clonally propagated by rooting of shoot cuttings and all sequence data was obtained from this single clone.

**DNA and RNA extraction**

Genomic DNA and total RNA for Illumina sequencing was prepared using the DNeasy and RNeasy Plant Mini Kits, respectively (Qiagen, Manchester, UK), according to the manufacturer’s instructions. High molecular weight genomic DNA for PacBio sequencing and for Bionano optical mapping was prepared by the Earlham Institure (previously known as The Genome Analysis Centre, Norwich, UK) using a Bionano Prep Plant Tissue DNA isolation kit, according to manufacturer’s instructions; this involved purification of nuclei which were then embedded in agarose and digested with proteinase K and RNase A before recovery of DNA with agarase.

**Sequencing data**

Two PCR-free Paired-End libraries with a read length of 250 base pairs (bp) and an insert size of 395 bp were prepared for sequencing on an Illumina Hiseq2500^TM^ platform at the Earlham Institute using the Whole Genome Sequencing approach. The sequencing yielded a total of ~172 Gbp and the quality of the Illumina reads was assessed with FastQC v0.11.

Long reads were sequenced on two different Pacific Bioscience platforms, RS-II and Sequel. ***RS-II****:* 18 Single Molecule, Real-Time cells were sequenced on a PacBio RS-II platform with P6-C4 chemistry. In total, ~8.7 Gbp of raw sequencing data were generated in ~1.3 million reads. The N50 of the reads was 10,991 bp. ***Sequel:*** 12 Single Molecule, Real-Time cells were sequenced on a Sequel Platform. In total, ~42.4 Gbp of raw sequencing data were generated in ~4.9 million reads. The reads N50 was 14,244 bp. The outputs from the two platforms were converted to fasta files and merged together for the subsequent analyses. The length distribution of the PacBio reads are Shown in Figure 1.

**A.** **
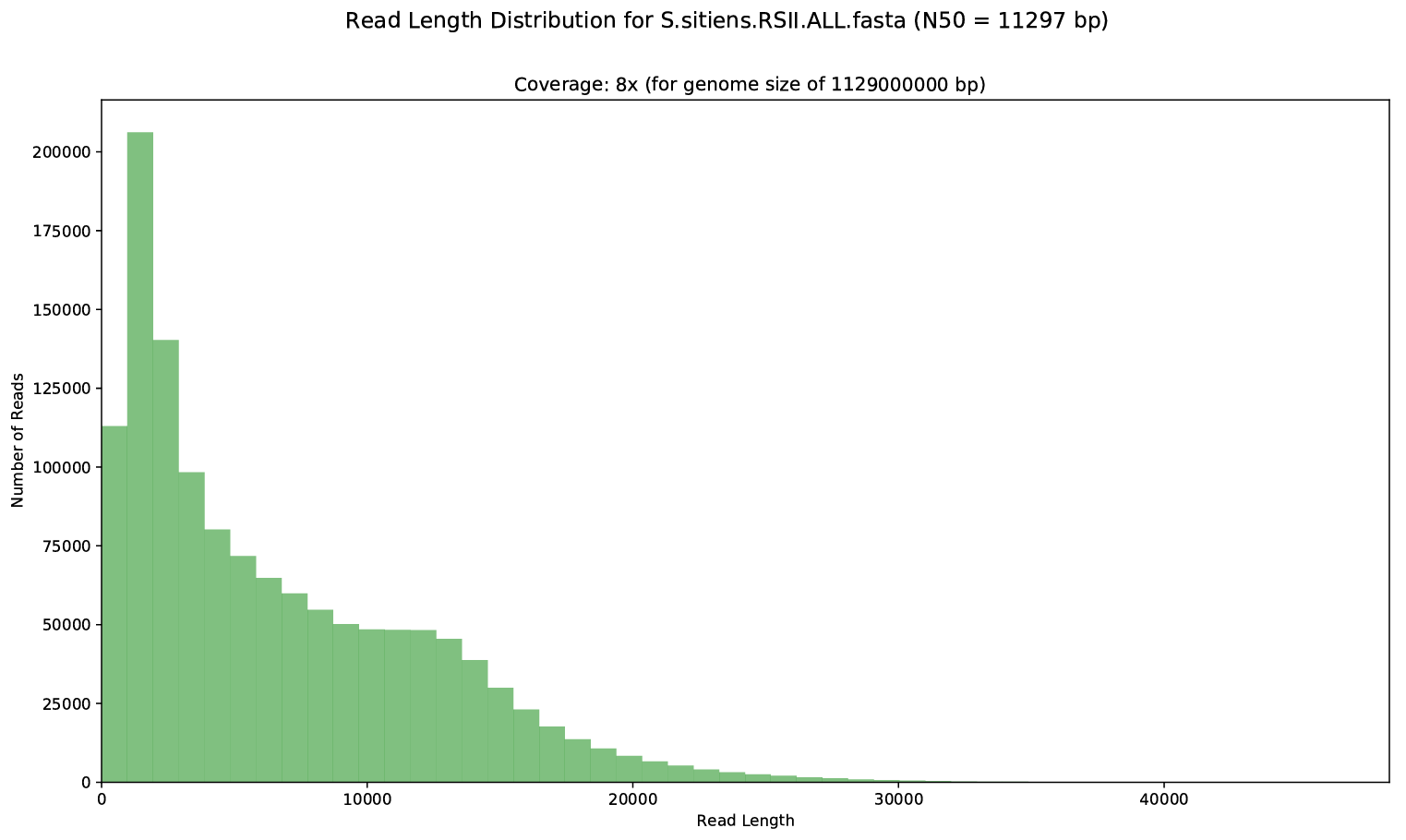
**

**B.** **
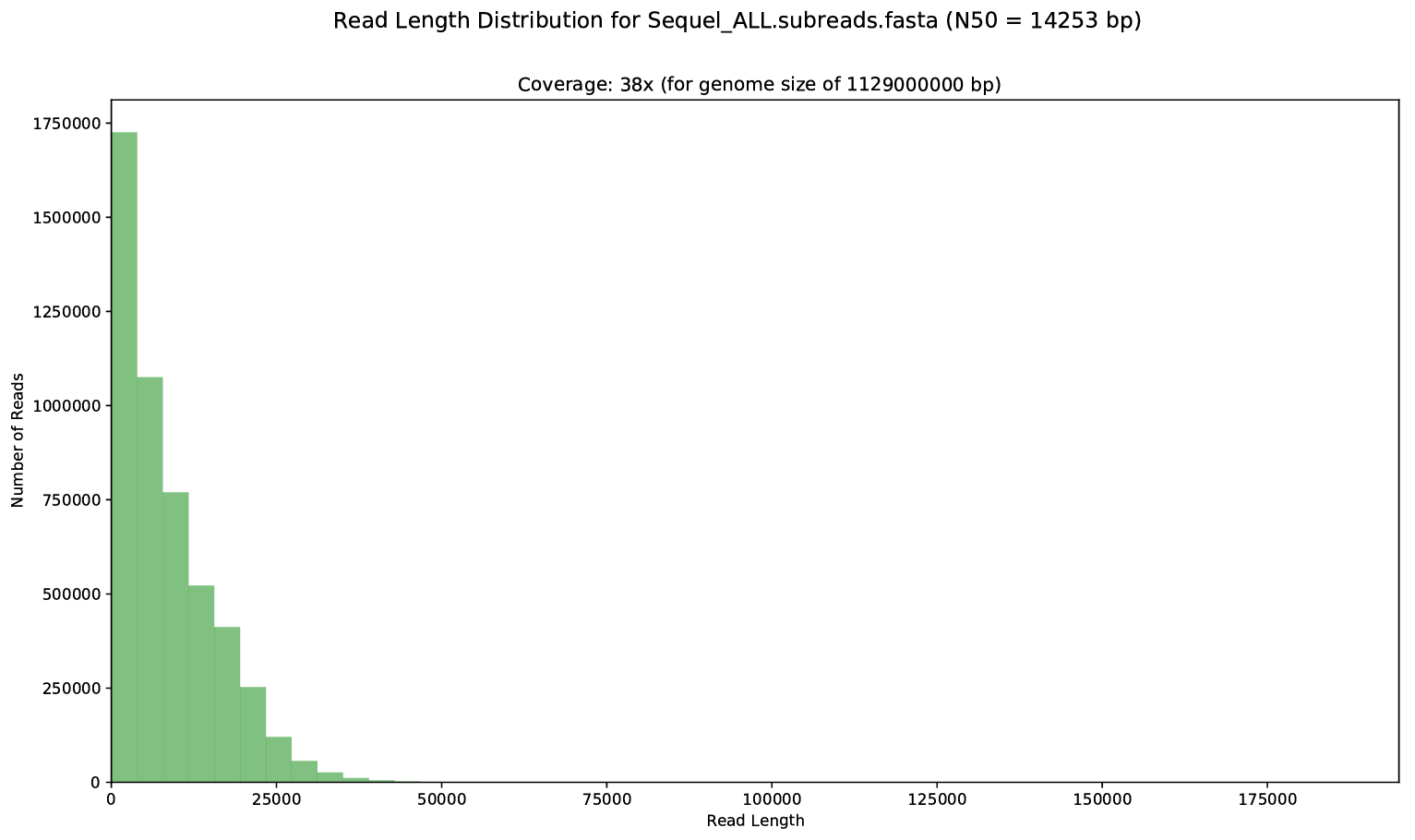
**

Figure 1: Length distribution of the Pacbio reads, filtered by length lower than 1 kbp A. the RSII platform B. the Sequel platform

Additionally, ~1,198 Gbp of optical maps longer than 100 kbp were generated on a BioNano Irys platform at the Earlham Institute using the BssS1 nicking enzyme. The raw bnx file was converted to cmap format and produced 4,426 consensus maps.

To assist in overcoming the heterozygosity challenge for *S. sitiens* and to generate phased haplotypes reference genome, a single library of Paired-End 10X Chromium data was sequenced from a fresh leaf at the Earlham Institute following 10x Genomics guidelines for genomes between 0.1 and 1.6 Gbp. The resulting fastq files contained ~40 Gbp of data. The *“basic”* pipeline from LongRanger v2.2.2 interleaved the two fastq files and performed read trimming, barcode error correction and barcode whitelisting. LongRanger also moved the 10x molecule barcode, present in the first 16 bp of each left read, to the corresponding pair read names, a necessary step for most downstream analyses. A custom Perl command (available at <https://github.com/MCorentin/Solanum_sitiens_assembly>) added the barcode to the read name to accommodate Arcs (Yeo et al. 2018) requirements. The reads without a barcode were removed, this filtered ~13 million reads, corresponding to 5% of the total amount.

12 Illumina Paired-End RNA-Seq libraries, generating a total of 63 Gbp of reads were sequenced, first to develop a *de novo* transcriptome assembly and second to generate hints for guided gene prediction from the genome assembly. The quality of the RNA-Seq reads was assessed with FastQC. Pair of reads with at least one read containing unfixable errors, as detected by Rcorrector (Song and Florea 2015), were removed from the assembly. Rcorrector utilises a k-mer spectra-based methodology to convert rare k-mers within the dataset to trusted k-mers which are more commonly observed within the reads. Here 23-mers were used. Rare k-mers are likely to represent sequencing errors and, although in some cases may be biologically real, were removed to prevent any adverse impact on assembly quality. Trimming of the adapters and low-quality ends from the reads, with a score lower than 5, was done with TrimGalore v0.6.0.

Detailed information on the sequencing throughput and statistics can be found in Tables ST1-5.

Table 1: Illumina sequencing statistics

| **Platform** | **Metric** | **Value** |
| --- | --- | --- |
| Illumina HiSeq2500 2 x 250 bp PCR-free library | Number of lanes | 2 |
|  | Mean insert size (bp) | 395 |
|  | Estimated Coverage (x fold)* | 152 |
|  | Number of reads for 1490_R1 | 173,312,317 |
|  | Number of reads for 1490_R2 | 173,312,317 |
|  | Number of reads for 1494_R1 | 170,292,744 |
|  | Number of reads for 1494_R2 | 170,292,744 |
|  | **Total number of reads** | **687,210,122** |
|  | **Total number of bases** | **171,802,530,500** |
|  |  |  |
| * Based on an estimated genome size of 1,129 Mbp | |  |

Table 2: Pacbio RS-II sequencing statistics

| **Pacbio RSII** | | | | | | |
| --- | --- | --- | --- | --- | --- | --- |
| **SMRT cell** | **Subread number** | | **Total bases** | | **Mean subread  size** | **Subread N50** |
| A02_1 | 31,486 | | 190,395,596 | | 6,046 | 10,403 |
| A07_1 | 91,617 | | 621,638,083 | | 6,785 | 11,516 |
| B02_1 | 22,146 | | 124,687,944 | | 5,630 | 9,767 |
| B07_1 | 92,144 | | 613,980,300 | | 6,663 | 11,470 |
| C02_1 | 33,233 | | 184,908,582 | | 5,564 | 9,768 |
| C07_1 | 88,545 | | 597,389,780 | | 6,746 | 11,438 |
| D02_1 | 28,558 | | 165,003,220 | | 5,777 | 10,191 |
| D07_1 | 87,407 | | 555,236,851 | | 6,352 | 11,035 |
| E06_1 | 102,198 | | 690,963,459 | | 6,761 | 11,381 |
| E07_1 | 88,112 | | 583,428,494 | | 6,621 | 11,181 |
| F06_1 | 123,518 | | 879,429,858 | | 7,119 | 11,560 |
| F07_1 | 92,187 | | 650,554,476 | | 7,056 | 11,564 |
| G01_1 | 29,399 | | 176,807,514 | | 6,014 | 10,371 |
| G06_1 | 69,938 | | 493,894,103 | | 7,061 | 11,469 |
| G07_1 | 93,175 | | 712,148,126 | | 7,643 | 11,798 |
| H01_1 | 32,092 | | 189,913,238 | | 5,917 | 10,349 |
| H06_1 | 86,587 | | 561,121,406 | | 6,480 | 11,282 |
| PSEQ-942_G06_1 | 109,510 | | 719,651,956 | | 6,571 | 11,296 |
| **TOTAL** | **1,301,852** | | **8,711,152,986** | |  |  |
|  |  | |  | |  |  |
| Number of SMRT cells | | 18 | |  |  |  |
| Average subread number | | 72,325 | |  |  |  |
| Average Mbp/cell | | 484 | |  |  |  |
| Average subread size | | 6,489 | |  |  |  |
| Average subread N50 | | 10,991 | |  |  |  |
| Coverage (x fold)* | | 8 | |  |  |  |
|  |  | |  | |  |  |
|  | * Based on an estimated genome size of 1,129 Mbp | |  | |  |  |

Table 3: Pacbio Sequel sequencing statistics

| **Pacbio Sequel** | | | | | | |
| --- | --- | --- | --- | --- | --- | --- |
| **SMRT cell** | **Subread number** | | **Total bases** | | **Mean subread  size** | **Subread N50** |
| PSEQ-1416_1_A01 | 230,475 | | 2,156,815,929 | | 9,358 | 15,307 |
| PSEQ-1416_2_B01 | 290,195 | | 2,456,357,738 | | 8,465 | 14,385 |
| PSEQ-1425_1_A01 | 498,204 | | 4,474,605,251 | | 8,981 | 15,045 |
| PSEQ-1425_2_B01 | 475,063 | | 3,991,010,408 | | 8,401 | 14,217 |
| PSEQ-1425_3_C01 | 549,304 | | 4,868,031,846 | | 8,862 | 14,790 |
| PSEQ-1425_4_D01 | 456,064 | | 4,240,129,654 | | 9,297 | 15,225 |
| PSEQ-1425_5_E01 | 585,237 | | 4,947,186,814 | | 8,453 | 14,353 |
| PSEQ-1425_6_F01 | 246,286 | | 2,365,452,654 | | 9,604 | 15,913 |
| PSEQ-1433_3_C01 | 382,280 | | 3,153,866,402 | | 8,250 | 13,248 |
| PSEQ-1433_4_D01 | 345,859 | | 2,480,289,058 | | 7,171 | 12,285 |
| PSEQ-1440_1_A01 | 447,859 | | 3,857,380,862 | | 8,613 | 13,599 |
| PSEQ-1440_2_B01 | 471,750 | | 3,449,153,552 | | 7,311 | 12,566 |
| **TOTAL** | **4,978,576** | | **42,440,280,168** | |  |  |
|  |  | |  | |  |  |
|  |  | |  | |  |  |
| Number of SMRT cells | | 12 | |  |  |  |
| Average subread number | | 414,881 | |  |  |  |
| Average Mbp/cell | | 3,537 | |  |  |  |
| Average subread size | | 8,564 | |  |  |  |
| Average subread N50 | | 14,244 | |  |  |  |
| Coverage (x fold)* | | 38 | |  |  |  |
|  | |  | |  |  |  |
| * Based on an estimated genome size of 1,129 Mbp | | | | |  |  |

Table 4: Bionano molecules statistics (enzyme: BssSI)

| **Length Bin** | **# of Molecules** | **Quantity (Gbp)** | | **Bin Mass Fraction (%)** | **Labels density (/100 kbp)** |
| --- | --- | --- | --- | --- | --- |
| >100 kbp | 7,839,755 | | 1,197.60 | 100% | 8.34 |
| >150 kbp | 2,881,693 | | 596.6 | 49% | 8.33 |
| >180 kbp | 1,557,852 | | 380.5 | 31% | 8.31 |
| >250 kbp | 420,197 | | 145.7 | 12% | 8.49 |
| >500 kbp | 30,490 | | 24 | 2% | 9.85 |
| 150-200 kbp | 1,836,141 | | 313.1 | 26% | 8.32 |

Table 5: Chromium 10x sequencing statistics

| **Number of raw reads** | 272,261,506 |
| --- | --- |
| **Read length (bp)** | 150 |
|  |  |
| **Barcodes in LongRanger whitelist** | 94.97% |
| **Number of reads with correct barcode** | 258,569,070 |
| **Number of bases** | 38,785,360,500 |
| **Coverage (x fold)*** | 34 |
|  |  |
| * Based on estimated length of 1,129 Mbp | |

**Genome size estimation**

The genome size was estimated with a k-mer based approach (Williams et al. 2013), Jellyfish v2.2.3 (Marcais and Kingsford 2011), with the “-C” option to consider both strands, counted the k-mers (k=25) present in the two Illumina libraries. The 172 billion bases from the reads generated 129 billion 25-mers. They were plotted as a histogram (see Figure 2) which showed two peaks: one at 49 coverage the other at 99, corresponding to the heterozygous and homozygous peaks respectively. The 18.6 billion low coverage 25-mers, below 24 coverage, were considered artefacts and not used as input for the genome size estimation. The remaining 25-mers divided by the homozygous coverage of 99 revealed an estimated genome size of 1,129 Mbp and a heterozygosity of 1.2%. Detailed statistics are available in Table ST6.


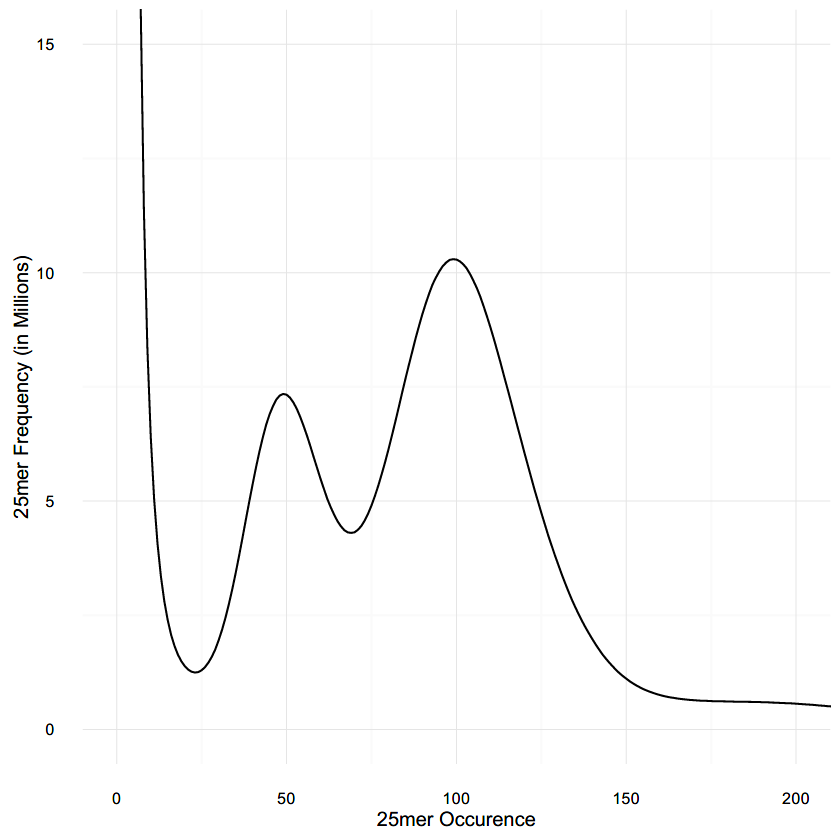


Figure 2: Histogram of the 25-mer distribution in the Illumina reads. Two peaks are visible, one at 49 the other at 99, corresponding to the heterozygous and homozygous peak respectively. The low frequency 25-mers, below 24, are considered artefacts.

Table 6: Genome size estimation with Jellyfish

| Illumina total bases: | 171,802,530,500 |
| --- | --- |
| k-mer size | 25 |
| Total number of kmers | 128,880,158,848 |
| Low occurence threshold: | 23 |
| Number of k-mer with occurence > 23 | 111,752,888,927 |
| Heterozygous peak: | 49 |
| **Homozygous peak:** | **99** |
| Heterozygous peak boundaries: | 24 - 69 |
| Homozygous peak boundaries: | 69 - 129 |
| **Estimated genome size (Mbp):** | **1,129** |
| **Estimated heterozygosity rate (%)** | **1.2** |

***De novo* genome assembly**

The contig assembly was generated with MaSuRCA v3.2.2 (Zimin et al. 2013), a hybrid assembler, taking as input both the Illumina and PacBio reads. Only the PacBio reads longer than 1 kbp were used, the Illumina reads were not trimmed, as advised in MaSuRCA’s documentation. For the deBruijn graph, the optimal k-mer size of 127 bp was automatically determined by MaSuRCA. The k-mer count threshold option was set to 2 as the Illumina coverage was expected to be more than 100x, the number of threads and the jellyfish hash size were set to 64 and 100 billion respectively. The default values were kept for the other parameters. (MaSuRCA config file is available at <https://github.com/MCorentin/Solanum_sitiens_assembly> “MaSuRCA/config.txt”).

The contigs were scaffolded with SSPACE v1-1 (Boetzer et al. 2011) using 40 threads, the default options and the PacBio reads longer than 1 kbp.

The scaffolds were super-scaffolded with “Hybrid Scaffold” from BioNano Genomics using information from the optical map. The conflict filter levels were set to “no filter” (-B 1 and -N 1), and the configuration xml file (-c) is available at <https://github.com/MCorentin/Solanum_sitiens_assembly>, “HybridScaffold/hybridScaffold_config.xml”. The cutting enzyme from this project, “BssSI” (CACGAG), was not supported by Hybrid Scaffold and was manually added to the list of available enzymes. The Hybrid Scaffold algorithm discarded all the unmapped scaffolds, damaging the assembly completeness and quality. Hence, the Perl script “hybridScaffold_finish_fasta.pl” script (Shelton et al. 2015) was used to reintegrate them into the assembly.

The polishing of the assembly with the Illumina reads was performed with Pilon v1.22 (Walker et al. 2014). Both Illumina libraries were aligned to the scaffolds with the Burrow-Wheeler Aligner (bwa) v0.7.15 using the mem algorithm with the default parameters. Some memory issues were met when running Pilon against the whole assembly, hence we developed a custom script to perform the polishing of the assembly by batches of sequences (source code available at: <https://github.com/MCorentin/run_pilon_batches.sh>).

The gaps were filled with GapFiller v1-10 (Boetzer and Pirovano 2012), both Illumina libraries were used and defined as having the Forward Reverse (FR) orientation and an insert size of 395 bp, with an standard deviation of 0.25, tolerating insert sizes between 296 and 493 bp. GapFiller was run on 20 threads with 10 iterations and the minimum number of overlapping bases with the edge of the gap was set to 20, the default values were kept for the other parameters.

Some duplications, due to the heterozygous nature of the sample, remained, as shown by the BUSCO results on the assembly (Table ST7). To remove duplicated scaffolds, the *dedupe.sh* script from BBmap v37.32 was run on 60 threads with the following parameters: the *storequality* option was set to false, the *absorbrc* and *touppercase* options were set to true. The scaffolds were considered duplicates if their identity was higher than 90% and they had a minimum overlap of 1000 bases. The allowance for substitutions and edits were determined empirically between a range of 1,000 and 60,000 and 500 and 7,000 respectively. The final values of 40,000 and 5,000 were chosen by assessment of the results with Quast, BUSCO and KAT. See Table ST8 for more details.

Table 7: Quast and BUSCO statistics at each step of the assembly

|  | **Assembly stastistics** | | | | | | | | **Busco (embryophyta set: 3,052 genes)** | | |
| --- | --- | --- | --- | --- | --- | --- | --- | --- | --- | --- | --- |
| **Stage** | **Length  (Mbp)** | **# contigs /scaffolds** | **Largest  scaffold (bp)** | **N50 (bp)** | **N75 (bp)** | **n >= N50** | **# N's /  100 kbp** | **GC %** | **Complete  (Single+Duplicated)** | **Fragmented** | **Missing** |
| MaSuRCA  (contigs) | 1,255 | 5,492 | 11,182,222 | 805,210 | 333,931 | 403 | 62 | 35.38 | 2,900 (2,794 + 106) | 78 | 74 |
| SSPACE | 1,262 | 3,516 | 11,182,222 | 979,616 | 447,890 | 341 | 563 | 35.38 | 2,899 (2,796 + 103) | 79 | 74 |
| Hybrid Scaffold | 1,275 | 3,355 | 14,576,794 | 1,183,603 | 513,175 | 274 | 1,518 | 35.38 | 2,896 (2,788 + 108) | 83 | 73 |
| Pilon | 1,274 | 3,355 | 14,564,673 | 1,183,602 | 513,133 | 274 | 1,506 | 35.38 | 2,900 (2,793 + 107) | 78 | 74 |
| Gapfiller | 1,274 | 3,355 | 14,565,231 | 1,184,430 | 513,133 | 274 | 1,423 | 35.38 | 2,899 (2,792 + 107) | 77 | 76 |
| Dedupe | 1,245 | 2,153 | 14,565,231 | 1,214,320 | 559,963 | 262 | 1,410 | 35.34 | 2,901 (2,806 + 95) | 77 | 74 |
| **Arcs + Links** | **1,245** | **1,483** | **22,444,909** | **1,826,367** | **869,529** | **186** | **1,411** | **35.34** | **2,898 (2,802 + 96)** | **81** | **73** |

Table 8: BBmap dedupe results for different parameters

|  | **BBmap dedupe parameters** | | | | **Effect on assembly** | | | | |
| --- | --- | --- | --- | --- | --- | --- | --- | --- | --- |
|  | **minoverlap  (bp)** | **minidentity (%)** | **maxsubs** | **maxedits** | **# scaffolds** | **(KAT) Estimated  completeness** | **Busco  (embryophyta set: 3,052 genes)** | | |
|  |  |  |  |  |  |  | **Complete  (single+duplicated)** | **Fragmented** | **Missing** |
| *sitiens gapfilled* | *NA* | *NA* | *NA* | *NA* | *3,355* | *97.48%* | *2,899 (2,792 + 107)* | *77* | *76* |
| Test1 | 200 | 95 | 500 | 500 | 3,160 | 97.48% | 2,899 (2,792 + 107) | 77 | 76 |
| Test2 | 1,000 | 90 | 1,000 | 1,000 | 3,056 | 97.47% | 2,899 (2,792 + 107) | 77 | 76 |
| Test4 | 1,000 | 90 | 10,000 | 2,500 | 2,804 | 97.46% | 2,898 (2,792 + 106) | 78 | 76 |
| Test5 | 1,000 | 90 | 20,000 | 2,500 | 2,530 | 97.43% | 2,899 (2,797 + 102) | 77 | 76 |
| Test6 | 1,000 | 90 | 30,000 | 4,000 | 2,311 | 97.38% | 2,900 (2,802 + 98) | 77 | 75 |
| **Test7** | **1,000** | **90** | **40,000** | **5,000** | **2,153** | **97.28%** | **2,901 (2,806 + 95)** | **77** | **74** |
| Test8 | 2,000 | 90 | 50,000 | 6,000 | 2,022 | 97.18% | 2,899 (2,807 + 92) | 78 | 75 |
| Test9 | 5,000 | 90 | 60,000 | 7,000 | 1,910 | 97.02% | 2,899 (2,808 + 91) | 78 | 75 |

The 10x Genomics Chromium data was used as a final step to super scaffold the assembly with the Arcs and Links (Warren et al. 2015) pipeline (available at <https://github.com/bcgsc/arcs>). The Chromium data was aligned to the deduped assembly with bwa v0.7.15 using the mem algorithm with the smart pairing option enabled (-p) to indicate the interleaved nature of the data and otherwise default parameters. First, Arcs v8.25, with default values and harnessing the long-rage information contained in the Chromium data, generated a graph with the scaffolds as nodes and evidence of links between the scaffolds as edges. This graph was transformed to a tsv file with the “makeTSVfile.py” script distributed with Arcs and given as input to Links v1.8.5 for the super scaffolding. An overview of the assembly pipeline is available as Figure 3.


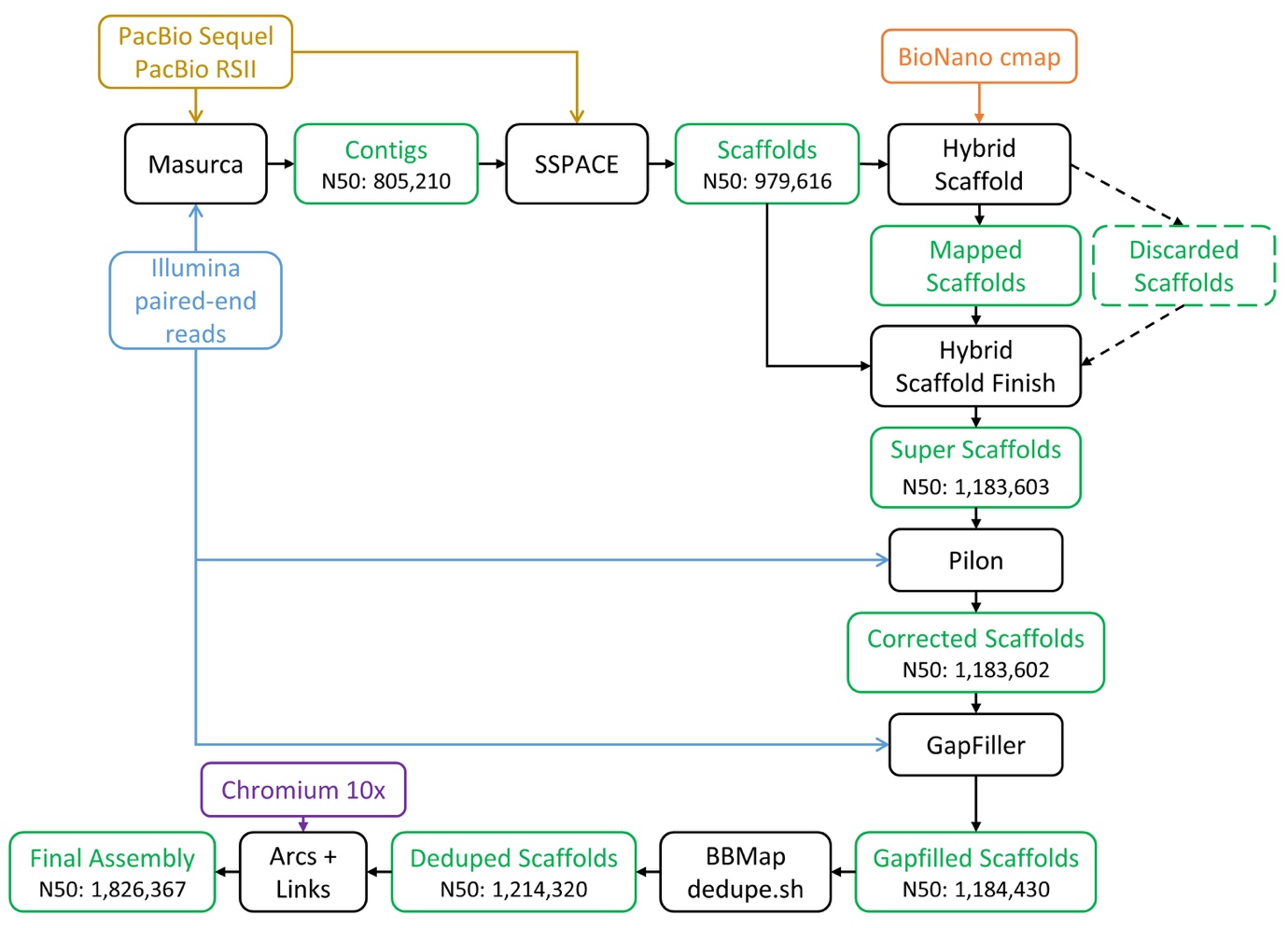


Figure 3: Overview of the assembly pipeline. Tools are in black, assembly steps in green.

**Assessment of the assembly quality**

At each step of the assembly process, results were assessed with Quast v4.5 (Gurevich et al. 2013) to measure the sequences length and contiguity, and BUSCO v3.0.2 (Simao et al. 2015) to estimate the completeness in term of gene content. For BUSCO, the “genome” mode was used to compare the assembly against the 3,052 benchmark genes from the OrthoDB v10 “Solanaceae” dataset(Zdobnov et al. 2017). To further confirm the final assembly completeness and duplication rate, KAT v2.4.0 (Mapleson et al. 2017) compared the 27mers presents in the two Illumina libraries against the 27mers from the assembly. Moreover, the RNA-seq data was mapped with STAR v2.6.0c (Dobin et al. 2013) to the draft assembly to validate the correctness and completeness with an independent dataset.

Contamination in the assembly was assessed by aligning the scaffolds, obtained after the GapFiller step, to NCBI’s Non-redundant Protein (NCBI-nr) database with Blastx-fast v2.3.0 (Altschul et al. 1990) with a word size of 20 and otherwise default parameters. The draft assembly was also anchored using Mummer v4.0 (Kurtz et al. 2004) to the *S. Lycopersicum* reference genome v3.0 and *S. pennellii*, two closely related species. The nucmer algorithm was used, keeping only the unique anchors in both the reference and query (--mum) and the minimum length of a cluster of matches (-c) was set to 300. The resulting alignment files were filtered with delta-filter only keeping 1-to-1 alignments (-l), to avoid cluttering the dot plots, which were made with mummerplot, using the -large and -layout options.

**Functional annotation and gene characterisation**

For the *de novo* transcriptome assembly, the Trinity suite v2.8.5 (Grabherr et al. 2011) was run with a k-mer size of 25 and a read coverage normalised to a maximum of 50. In order to minimise the time and computational resources required to analyse and annotate the assembly a number of methods to reduce duplication and filter out sequencing artefacts presenting as lowly expressed transcripts were performed and compared. Presently, the homologous transcripts, with an identity higher than 95%, were clustered by CD-HIT (Li and Godzik 2006) and duplicates removed, in addition the transcripts with an expression lower than 1.5 Transcripts Per Million (TPM) were filtered. The transcriptome assembly quality metrics were measured with the Trinity Perl script “TrinityStats.pl”, the completeness was confirmed using BUSCO against the *Solanaceae* OrthoDB10, containing 3,502 single copy benchmark genes.

In parallel, guided gene prediction with Augustus v3.3.2 (Stanke and Morgenstern 2005) was done on the genome assembly. First, RepeatMasker v4.0.9 (Smit et al. 2013) soft-masked the repeats, using “tomato” as species and a library of repeats, “[draft_repeats_master.v5.fasta](ftp://ftp.solgenomics.net/tomato_genome/repeats/draft_repeats_master.v5.fasta)”, available at <ftp://ftp.solgenomics.net/tomato_genome/repeats/>. STAR v2.6.0c was used to align the RNA-Seq reads to the masked assembly with intron hints extracted from the resulting BAM file into a GFF file via Augustus’ bam2hints.pl script. Whilst it is possible to extract exon locations to further aid gene prediction, reads may also align to UTRs and thus, due to a lack of trained UTR parameters for *S. sitiens,* this was not performed. Augustus was run using parameters trained for *S. lycopersicum* with repeat masking and intron hints as extrinsic evidence; default hints weighting was used.

Functional annotations of the transcriptome and gene prediction was performed with OmicsBox v1.1.164 (Conesa and Gotz 2008), using a local BLASTX-fast installation with default parameters against NCBI-nr and custom databases produced from Sol genomics’ *S. lycopersicum* (ITAG3.2) and *S. pennellii* (Spenn) (Fernandez-Pozo et al. 2015) annotated proteins, and The Arabidopsis Information Resources’ (TAIR10) (Berardini et al. 2015). The blast top-hit results were then associated with Gene Ontology (GO) terms and enzyme annotations. Moreover, protein domains in the transcriptome were identified with an InterProScan (Jones et al. 2014) search against the following databases, CDD, HAMAP, HMMPanther, HMMPfam, HMMPIR, FPrintScan, ProfileScan and HMMTigr.

For the comparative orthology, protein sequences were extracted from the *S. sitiens* and *S. chilense* gene predictions (unpublished data) GFF output files via Augustus’s Gff2aa.pl script. These protein sequences in addition to sequences from *S. pimpinellifolium, S. lycopersicum, S. pennellii, S. tuberosum* (Sol Genomics) and *A. thaliana* (TAIR10) were entered into OrthoFinder v2.3.3 (Emms and Kelly 2015) for the identification of common orthogroups and putative orthologues between each of the species through an all vs all DIAMOND BLAST algorithm. The resulting rooted species tree was plotted using GGtree (Yu et al. 2018). Predicted proteins assigned to orthogroups unique to *S. sitiens* were blasted against the NCBI-nr database and functionally annotated facilitating identification of stress-relevant putative proteins.

**Pseudomolecule assemblies based on *S. lycopersicum* and *S. pennellii***

The script *chromosome_scaffolder.sh* from MaSuRCA was launched to generate two *S. sitiens* pseudomolecule assemblies, based on two different references, *S. lycopersicum v3.0* and *S. pennellii*. The pseudomolecules are generated by first splitting the scaffolds into contigs, which are then aligned to the reference sequences with blasr. For both references, the sequence similarity threshold (-i option) was set to 80, and the PacBio RSII reads were aligned to the reference to check for mis-assemblies (-s option).

**Supplementary Materials – S2: Supplementary Tables**

Table ST 1: Assembly completeness and heterozygous rate assessment with KAT

| K-value used: | 27 |
| --- | --- |
|  |  |
| Total 27mers in the Illumina reads: | 154,064,638,327 |
| Total 27mers in the assembly: | 1,227,344,907 |
|  |  |
| Distinct 27mers in the Illumina reads: | 16,562,792,381 |
| Distinct 27mers in the assembly: | 740,579,624 |
|  |  |
| Distinct 27mers only found in the Illumina reads: | 15,824,014,361 |
| Distinct 27mers only found in the assembly: | 1,801,604 |
|  |  |
| Shared 27mers found in the Illumina reads: | 128,147,318,718 |
| Shared 27mers found in the assembly: | 1,225,373,114 |
|  |  |
| Peaks in the analysis: | 3 |
| Estimated genome size: | 841.37 Mbp |
| **Estimated heterozygous rate:** | **1.19%** |
| **Estimated assembly completeness:** | **97.28%** |

Table ST 2: BUSCO comparison between different assemblies

|  | **Busco (embryophyta set: 3,052 genes)** | | |
| --- | --- | --- | --- |
| **Assembly** | **Complete (Single+Duplicated)** | **Fragmented** | **Missing** |
| *S. sitiens* | 2,898 (2,802 + 96) | 81 | 73 |
| *S. lycopersicum v3* | 2,950 (2,923 + 27) | 48 | 54 |
| *S. pennellii* | 2,957 (2,936 + 21) | 40 | 55 |
| *S. pimpenellifolium* | 2,880 (2,820 + 60) | 80 | 92 |

Table ST 3: S. sitiens filtered de novo transcriptome assembly statistics

| Total length | 110,939,617 |
| --- | --- |
| Total Trinity 'genes' | 111,485 |
| Total Trinity transcripts | 131,581 |
| % GC | 40.91 |
| Contig N50 | 1,905 |
| Average contig length | 843 |
| Median contig length | 264 |

Table ST 4: List of genes related to drought and salt tolerance in the inversions against S. lycopersicum. The genes were obtained from the gene prediction done on the assembly with Augustus. The Solyc ID were obtained by blasting the predicted gene against the ITAG 4.0 annotation (ITAG4.0_gene_models.gff). The distance to the nearest breakpoint is an estimate based on the dotplots, the genes located outside an inversion have a negative value. A. Inversion on Scaffold95 B. Inversion on Scaffold8 C. Inversion on Scaffold11

A.

| **Scaffold95:800000 - 1120000** | | | |
| --- | --- | --- | --- |
| **Blast hit (ITAG4.0)** | **Distance to nearest breakpoint (kbp)** | **Gene name** | **Literature** |
| Solyc11g069960 | -31 | receptor‐like kinase RLK‐1 | *RLK1* is involved in response to salinity and drought stresses by activating or participating in Abscisic Acid (ABA) signaling (Osakabe et al. 2005; Zhao et al. 2013). |
| Solyc11g069940 | -5 | Glutaredoxin | In tomato, *SlGRX1* silencing increased the plant sensitivity to both drought and salt stresses (Guo et al., 2010). In cassava cultivars and maize, *CC type glutaredoxins* have been shown to be associated with drought response (Ding et al. 2019; Ruan et al. 2018). |
| Solyc11g069690 | 32 | protein disulfide-isomerase 5-1 | In *A. thaliana*, *PDI5* is involved in drought signaling (Kumar et al. 2015). Moreover, members of this protein family (*PDI*) are improving protein folding and transport during stress (Zhu et al. 2014). |
| Solyc11g069700 | 42 | elongation factor 1-alpha | In soybean, *EF1α* genes were upregulated by ABA, salt and drought stresses (Gao et al. 2019). |
| Solyc11g069735 | 80 | High-affinity nitrate transporter 2.2 | Higher expression of *NRT2.1* and *NRT2.2* was revealed to enhance drought resistance in *A. thaliana* (Zhong et al. 2015). |
| Solyc11g069765 | 111 | SNF1-related protein kinase regulatory subunit beta-3 | In plants, some members of the SNF1/AMP-activated kinases are activated by dehydration or ABA (Bartels and Sunkar 2005). |
| Solyc11g069770 | 130 | agamous-like MADS-box protein AGL62 | Considered as a top candidate gene for a “Water Use Efficiency” in dry condition QTL in *A. thaliana* (Lovell et al. 2015). |
| Solyc11g069800 | 123 | Allene oxide synthase, chloroplastic | *AOS* is an important enzyme in the jasmonic acid pathway, which plays a role in the plant response to salt stress. *AOS* accumulated in salt tolerant tomato plants after 6 hours of NaCl and jasmonic acid treatment (Pedranzani et al. 2003). In sweet potato and Arabidopsis*,* *AOS* was upregulated under salt stress (Jiang and Deyholos 2006; Zhang et al. 2017). |
| Solyc11g069820 | 45 | ABC transporter-like | Members of this family annotated with GO terms “response to salt stress” (GO:0009651) and “response to abscisic acid” (GO:0009737).  Overexpression of *AtABCG36* led to improved drought and salt tolerance in *Arabidopsis* (Kim et al., 2010). |
| Solyc11g069910 | 21 | DNA-directed RNA polymerase II subunit RPB11 | In *Arabidopsis,* RNA polymerase II quickly acted on drought-responsive genes when subjected to drought stress and rapidly disappeared during rehydration(Kim et al. 2010). |

**B.**

| **Scaffold8:2250000-2430000** | | | |
| --- | --- | --- | --- |
| **Blast hit (ITAG4.0)** | **Distance to nearest breakpoint (kbp)** | **Gene name** | **Literature** |
| Solyc09g091170 | -8 | NADH dehydrogenase | In *Nicotiana tabacum,* two NADH dehydrogenases are over expressed during drought stress (Xie et al. 2016). *AtNDB2*, an Arabidopsis NADH dehydrogenase was identified as being important for tolerance to environmental stress. Plants lacking *atNCB2* were less tolerant to drought and elevated light treatments (Sweetman et al. 2019). |
| Solyc09g091030 | 19 | Beta-amylase | *BAM1* is annotated with the GO Term “response to water deprivation” (GO:0009414).  *BAM1* is involved in the starch/proline interplay, which has been identified as a candidate trait to improve drought tolerance in crops (Zanella et al. 2016). Drought tolerance was improved in an *A. thaliana* *BAM1* mutant, because the resulting decrease in starch breakdown led to reduced stomatal opening (Prasch et al. 2015). |
| Solyc09g091060 | 44 | golgin subfamily A member 6-like protein 2 | This gene has been reported as up-regulated in the leaves of UCB-1 pistachio rootstock under drought stress, with a probable role in transportation and glycosylation of lipids and proteins under water deficit (Pakzad et al. 2019). |
| Solyc09g091090 | 66 | probable indole-3-pyruvate monooxygenase YUCCA7 | *YUCCA7* is annotated with the GO Term “response to water deprivation” (GO:0009414).   From the TAIR10 description ([AT2G33230.1](https://www.arabidopsis.org/servlets/TairObject?type=gene&id=33919)): “Encodes a flavin monooxygenase gene which belongs to the tryptophan-dependent auxin biosynthetic pathway and enhances drought resistance.”  In *Arabidopsis*, activation of *YUCCA7* led to increased drought tolerance (M. Lee et al. 2012). In rice, this gene is involved in auxin production, which increase root development and makes the plant more adapted to drought conditions (Wang et al. 2018). |
| Solyc09g091120 | 47 | BLISTER | *BLISTER* was shown to promote resistance to cold and drought stresses, by repressing ABA-reponsive PcG target genes in *A. thaliana*  (Kleinmanns et al. 2017). |
| Solyc09g090980 | -3 | pathogenesis-related protein STH-2-like (alternative name: PR10) | *SmPR10* expression in transgenic *Arabidopsis* was up-regulated, specific to roots and improved the plants tolerance to salt stress (Han et al. 2017). |
| Solyc09g090920 | -29 | Calcium-dependent lipid-binding (CaLB domain) family protein | In *A. thaliana,* loss of function of *AtCLB* increased the plant’s tolerance to salt and drought stresses (de Silva et al. 2011). |

**C.**

| **Scaffold11:5030000-5160000** | | | |
| --- | --- | --- | --- |
| **Blast hit (ITAG4.0)** | **Distance to nearest breakpoint (kbp)** | **Gene name** | **Literature** |
| Solyc10g079750 | -33 | BTB/POZ ankyrin repeat protein | *BOP1* and *BOP2* are necessary for cauline leaf abscission in response to drought in *Arabidopsis* (Patharkar and Walker 2016). |
| Solyc10g079600 | 8 | Two-component response regulator ARR9 | In *Arabidopsis*, *ARR9* was suggested to have a complex role, both positive and negative, in osmotic stress regulation (Wohlbach et al. 2008). |
| Solyc10g079580 | -5 | RNA-binding (RRM/RBD/RNP motifs) family protein | RNA-binding mechanisms might have roles in drought and salt stress response (K. Lee and Kang 2016; Marondedze et al. 2019). |

**Supplementary Materials – S3: Supplementary Figures**

**A.**


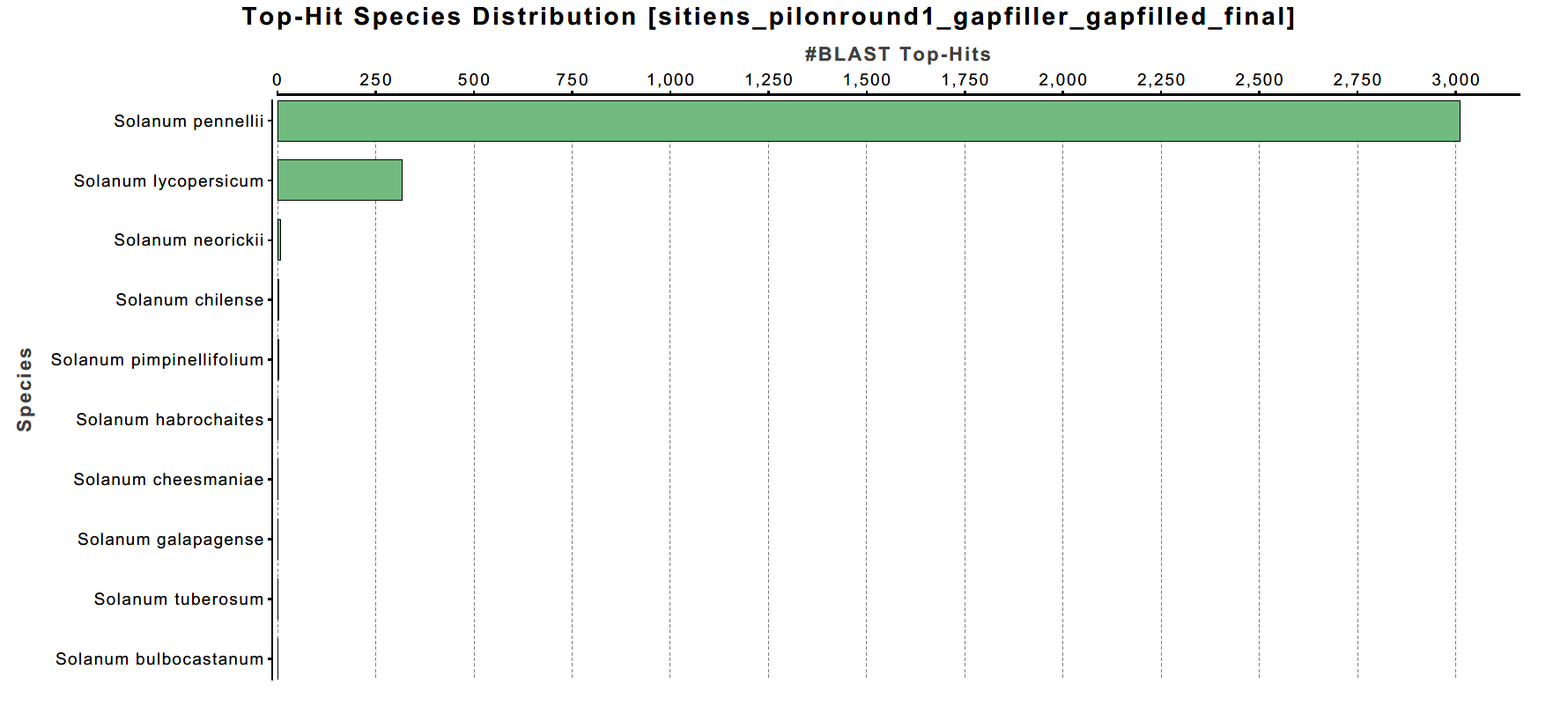


**B.**


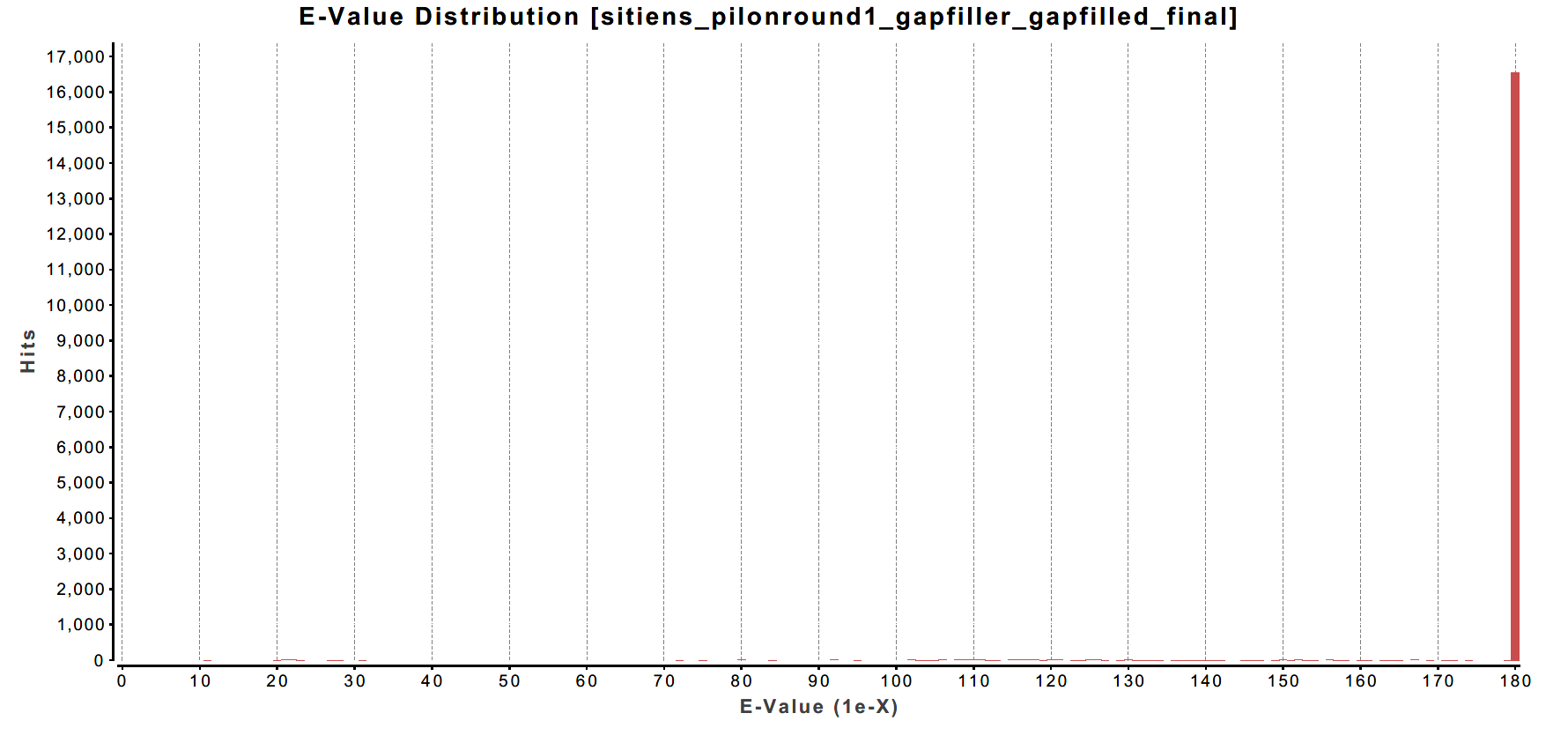


Figure SF 1: Results from the blast search of the scaffolds against NR. A. Distribution of the species for the blast top hit, consisting only of Solanum species as expected. B. Distribution of the e-value for all the hits, most of the hits have an e-value < 1e-180.


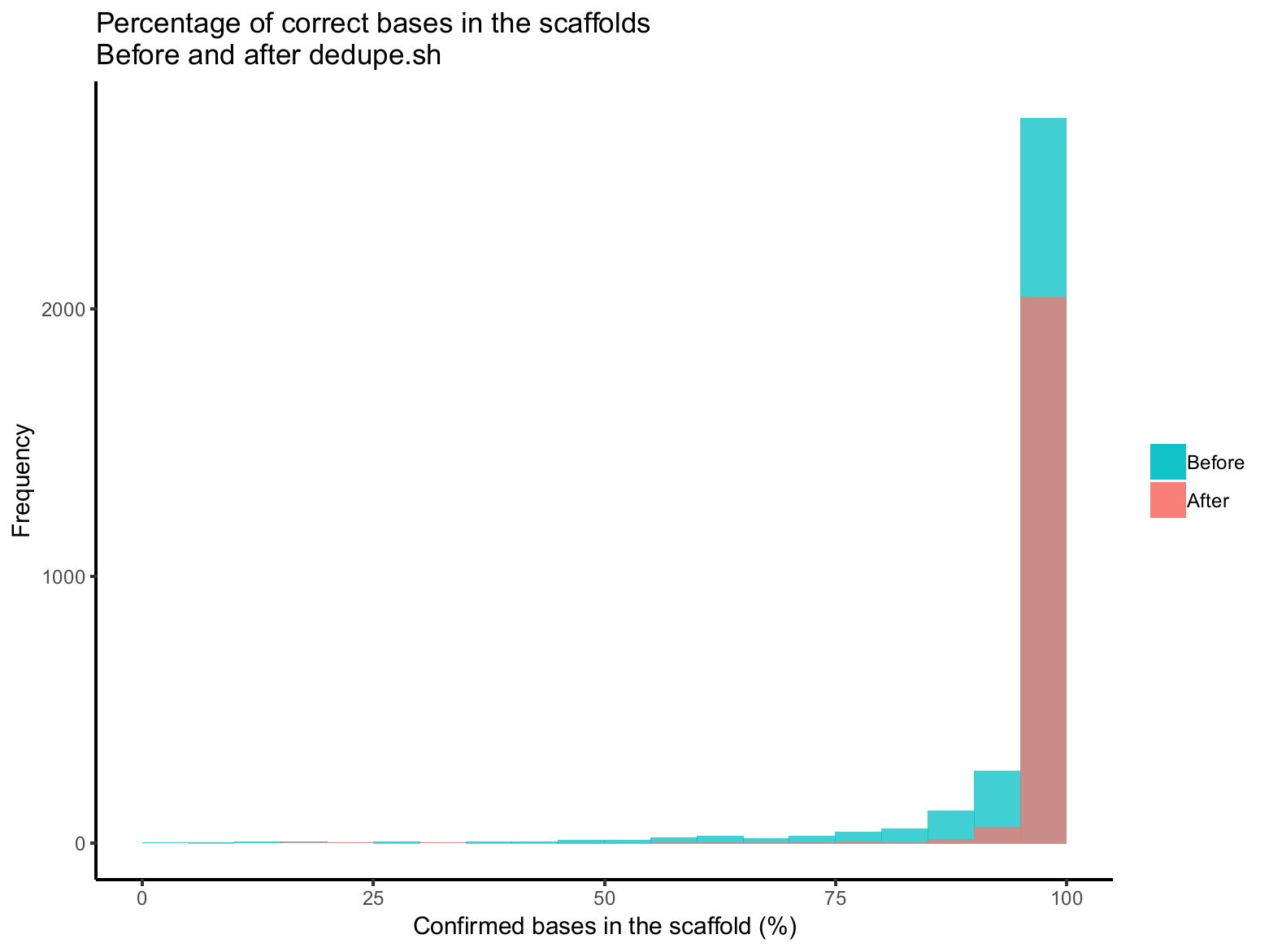


Figure SF 2: Pilon estimation of the correct bases in the assembly, based on the Illumina reads, before and after dedupe. The x-axis represents the percentage of correct bases, the y-axis represents the number of scaffolds. Most of the scaffolds with a percentage o


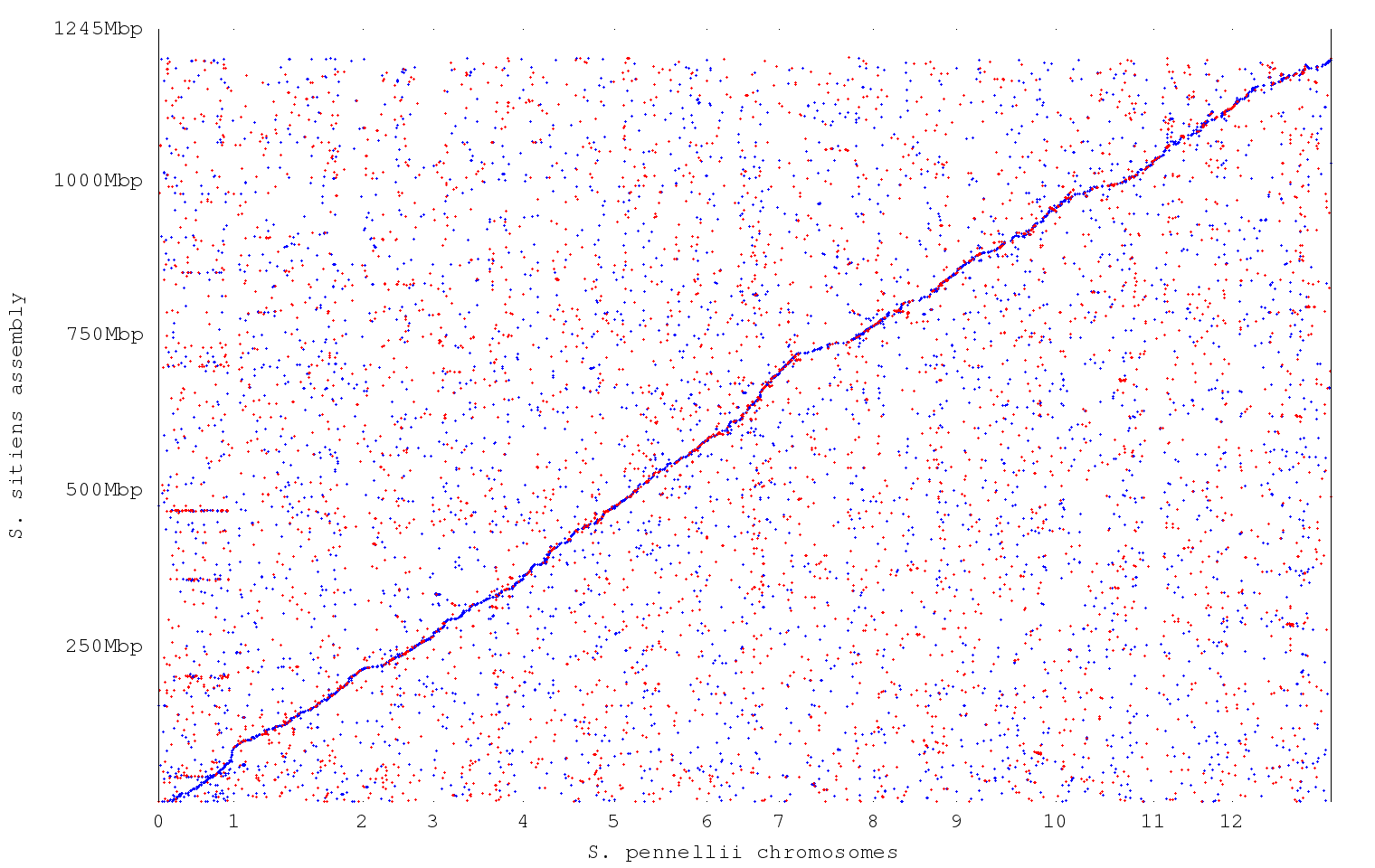


Figure SF 3: Dotplot of the similarity between the S sitiens assembly and the reference genome of S. pennellii, made with Mummer v4.00. Blue dots represent local forward alignments, red dots represent local reverse alignments.

**1A.**


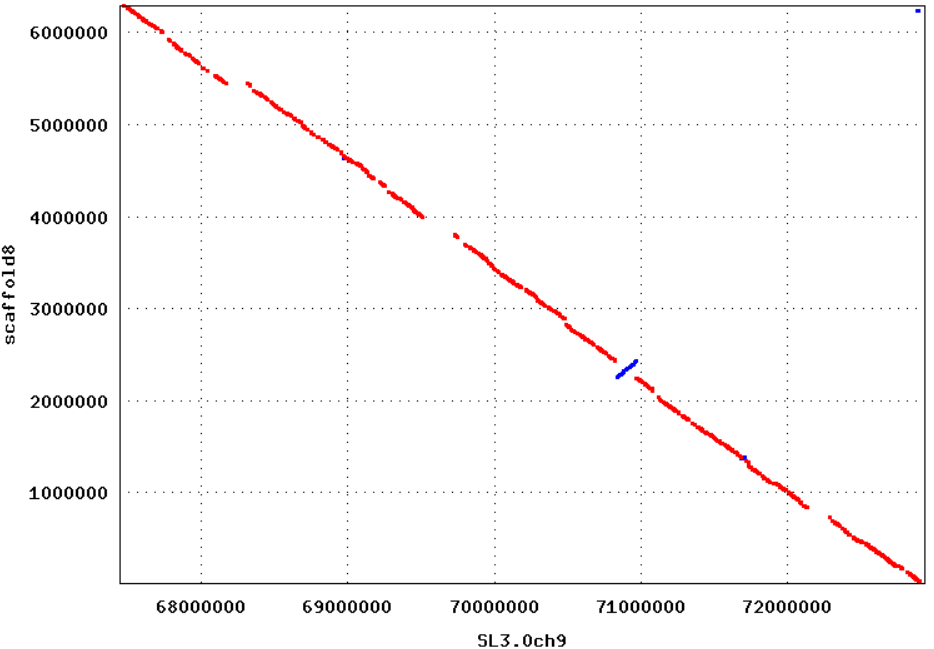


**1B.**


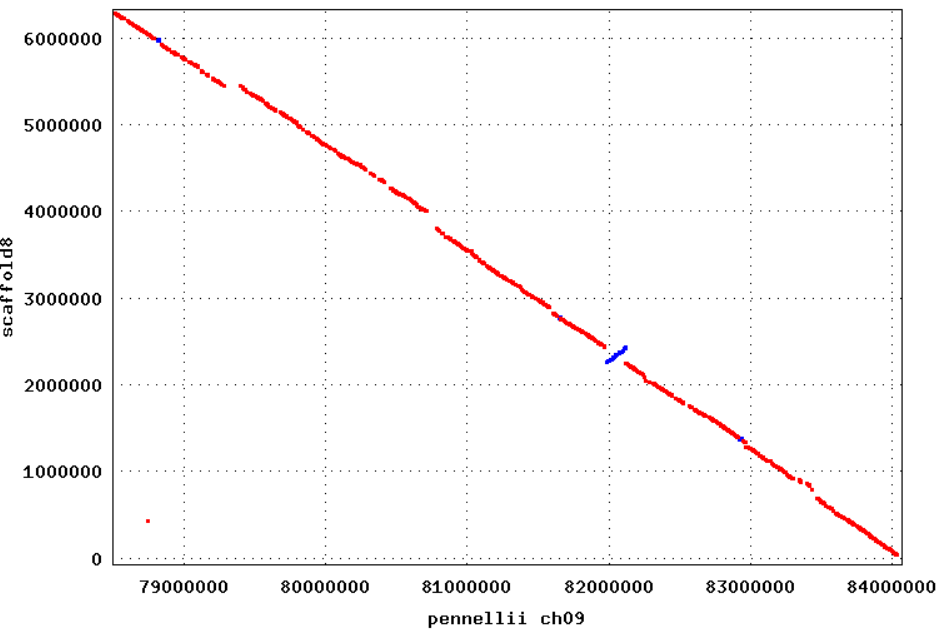
**2A**
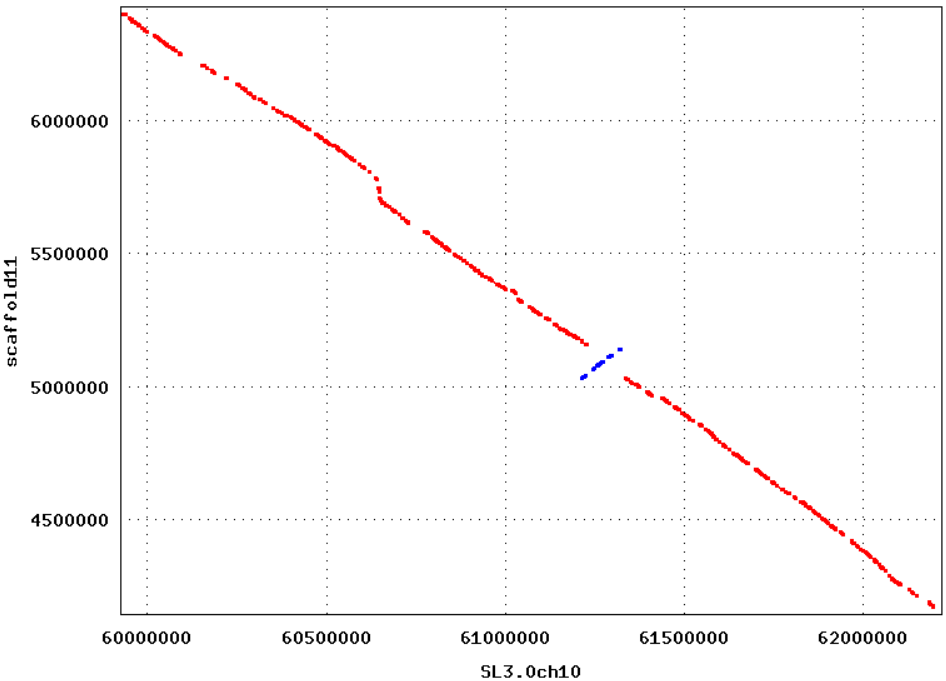


**2B.**
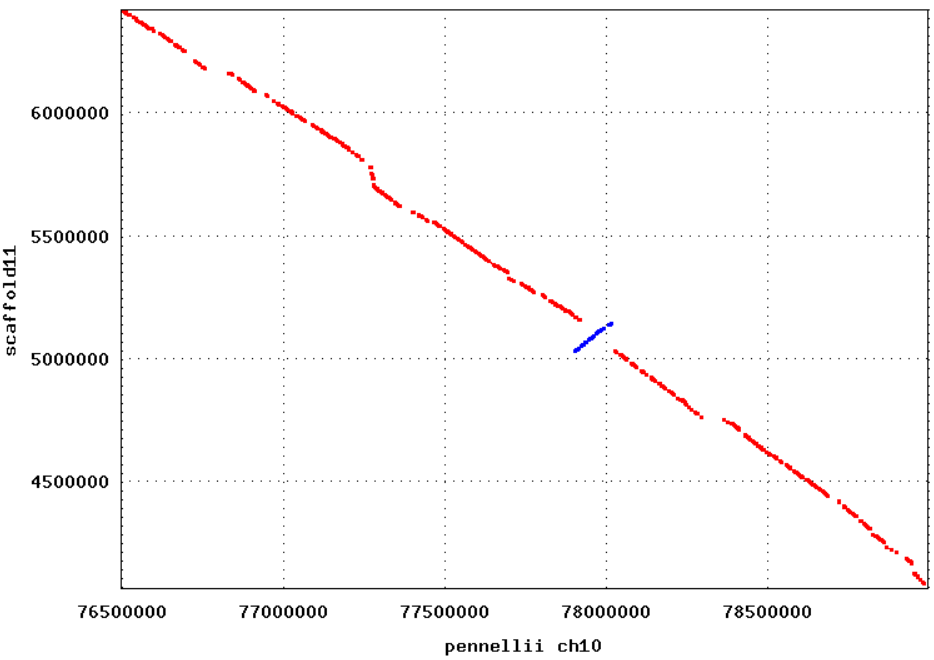
**3A.**


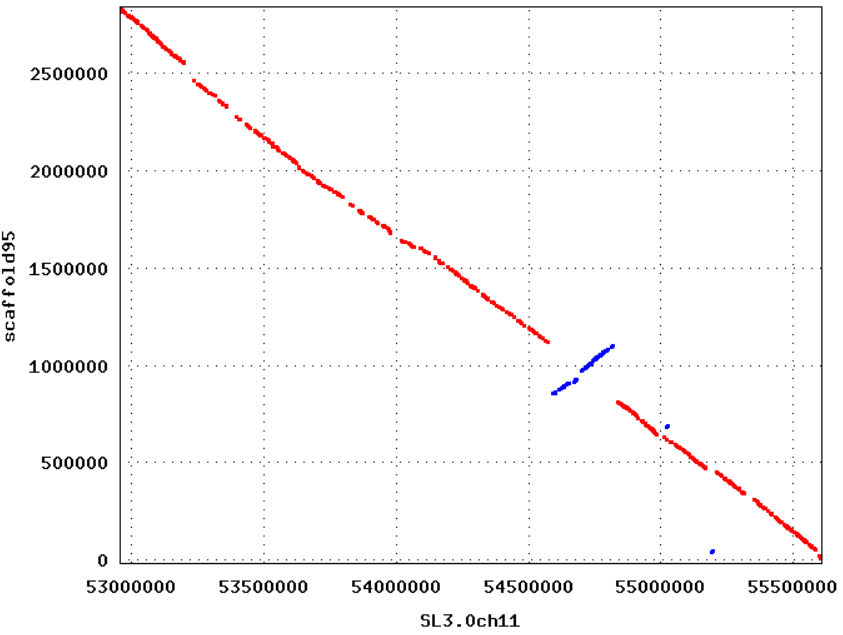


**3B.**
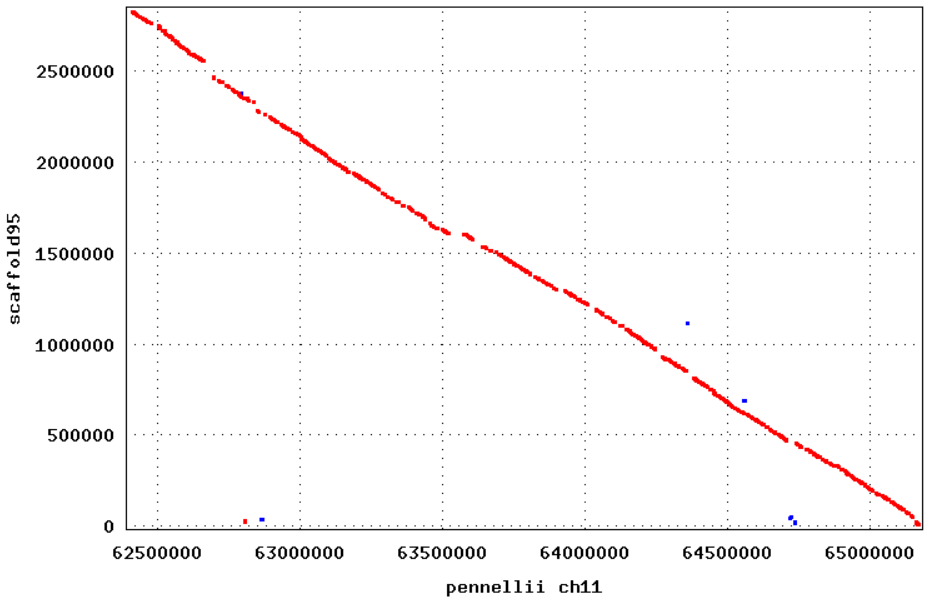


Figure SF 4: Dotplots of the S. sitiens inversions against S. lycopersicum v3.0 and S pennelli, done with Mummer v4. The sequences were aligned with nucmer, using the “—mum” and “-c 500” options. The dotplots were generated with mummerplot using the “-large” option.

**A.**

**
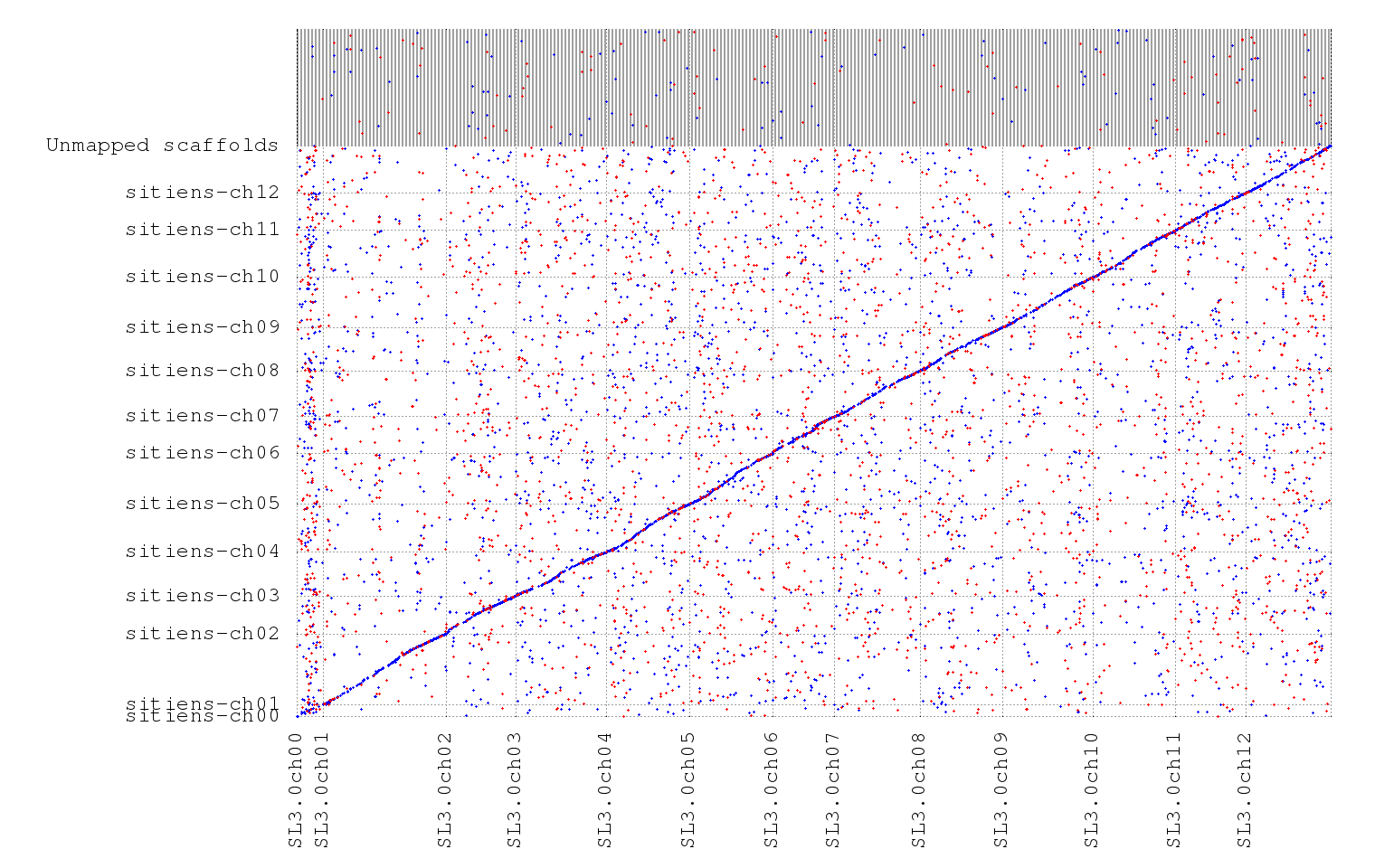
**

**B.** **
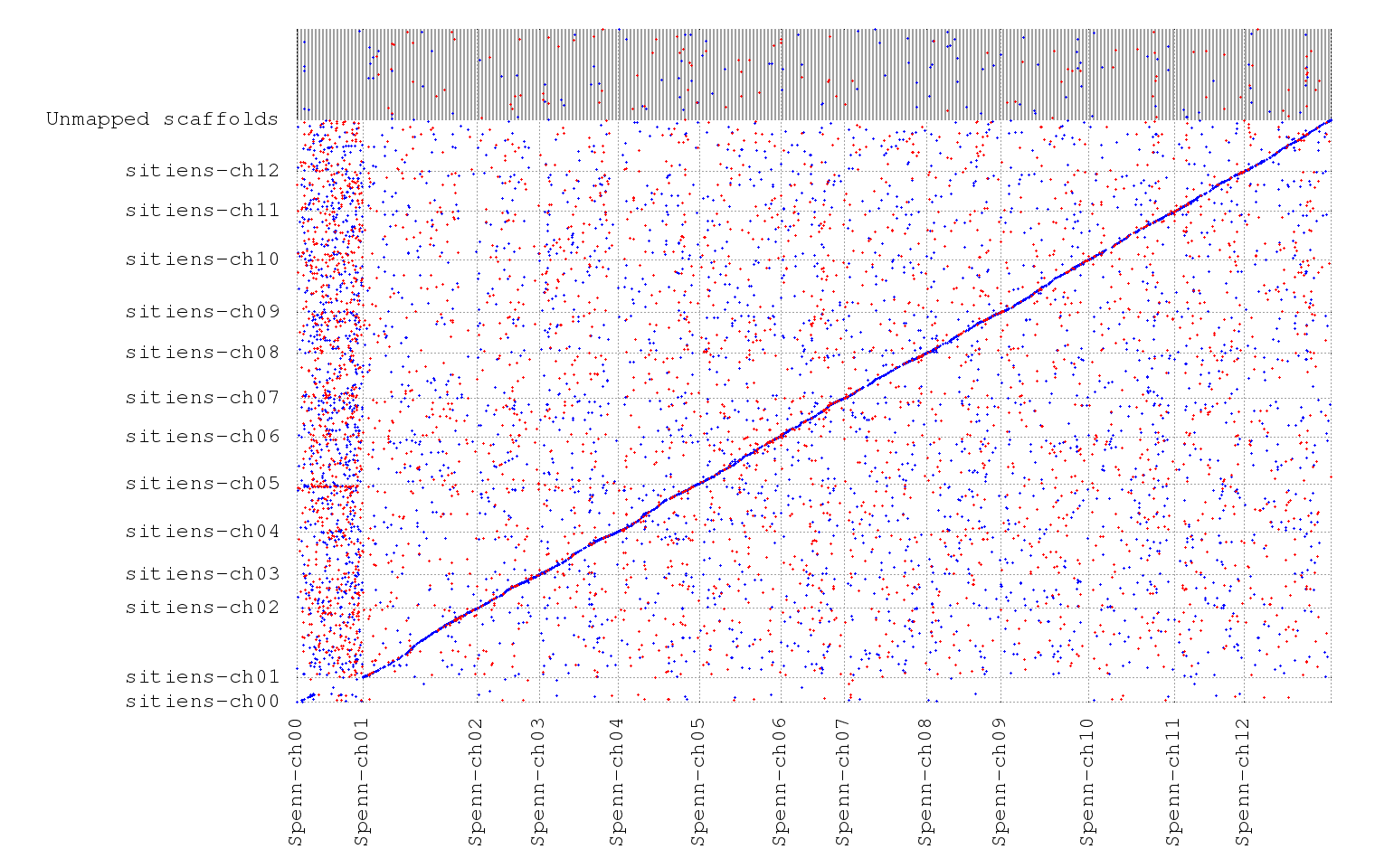
**

Figure SF 5: Dotplots of the S. sitiens pseudomolecules assemblies against their respective references used in the chromosome_scaffolder script. The assemblies were aligned with Mummer v4 using the “—mum” and “-c 500” options. The dotplots were generated with mummerplot using the “-large” option. A. Alignment against S. lycopersicum v3.0 B. Alignment against S pennelli.

**1A.
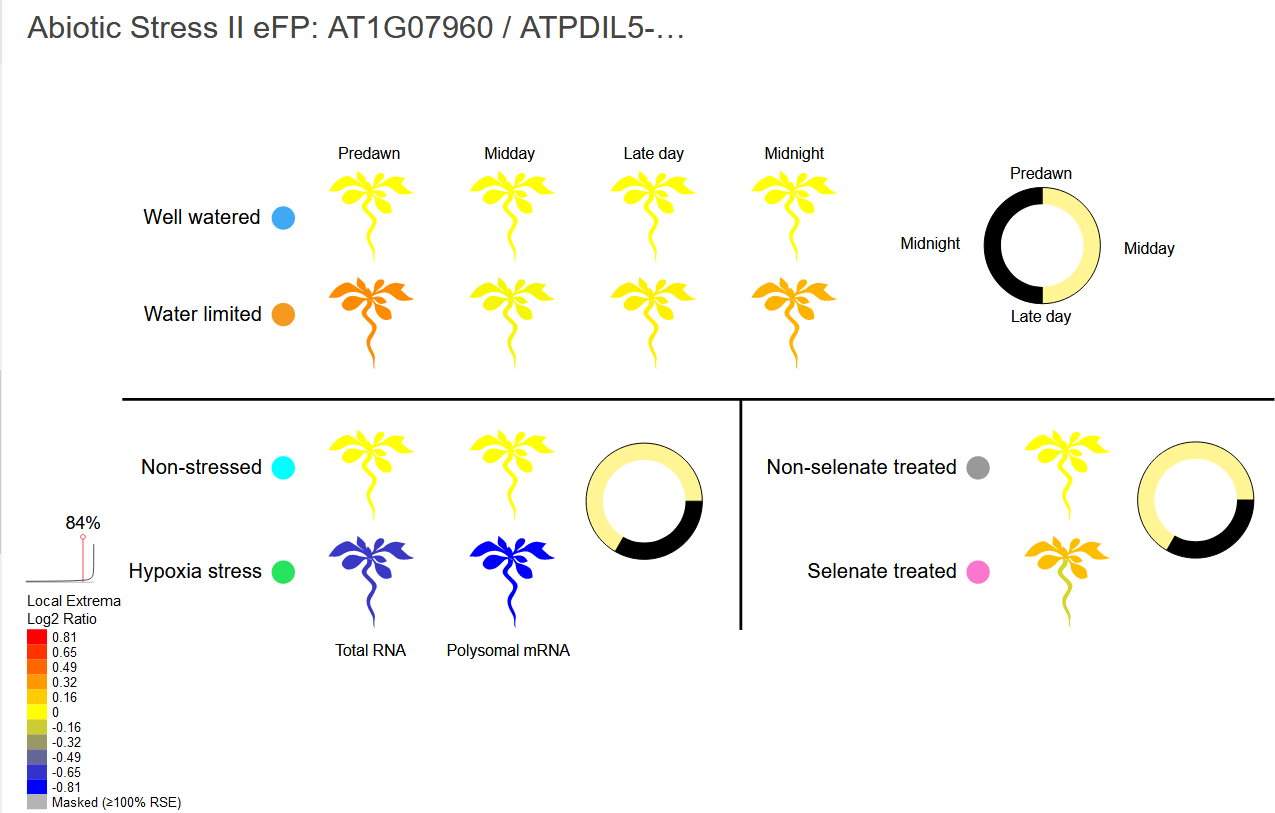
**


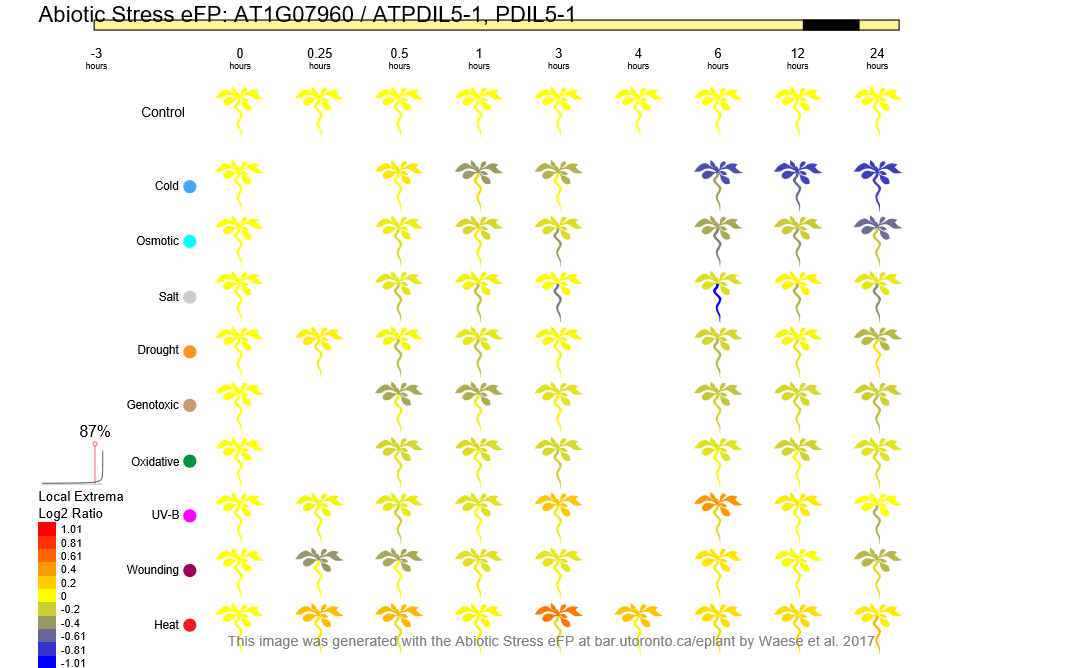
**1B.**

**2A.**


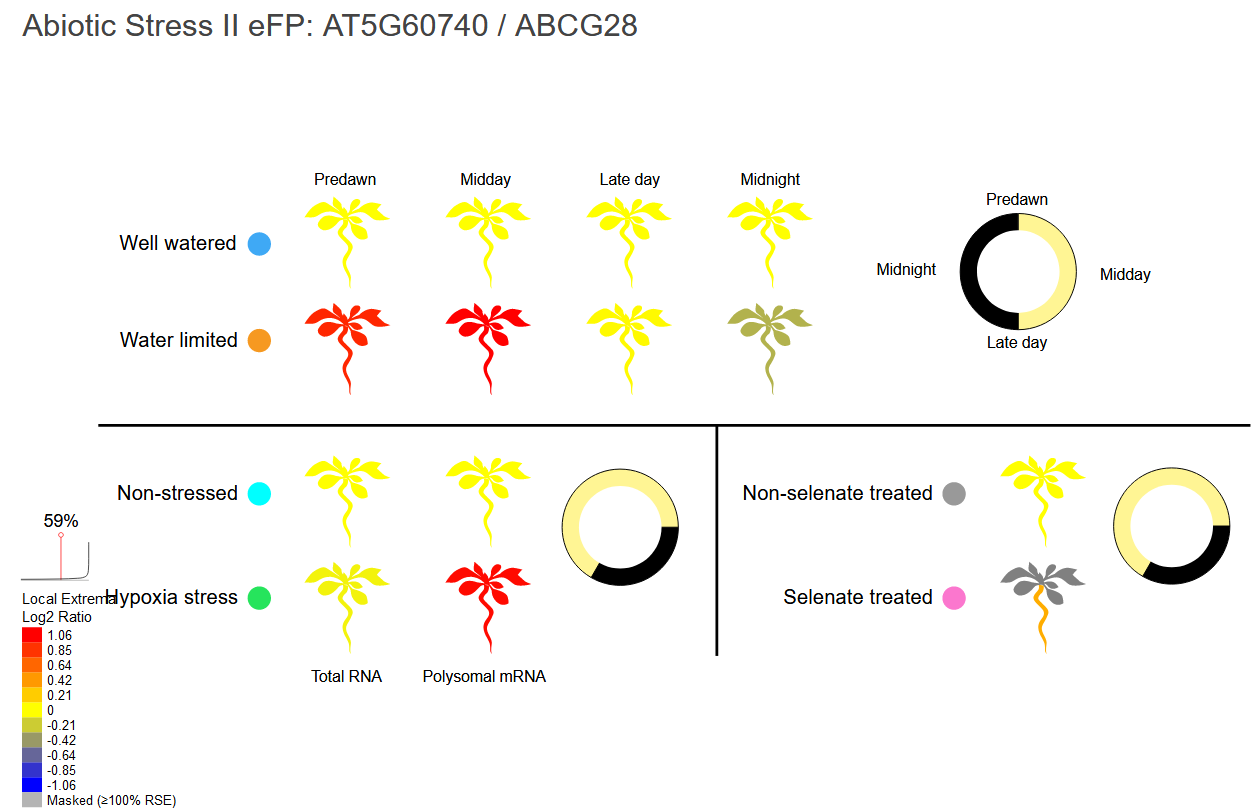


**2B.**


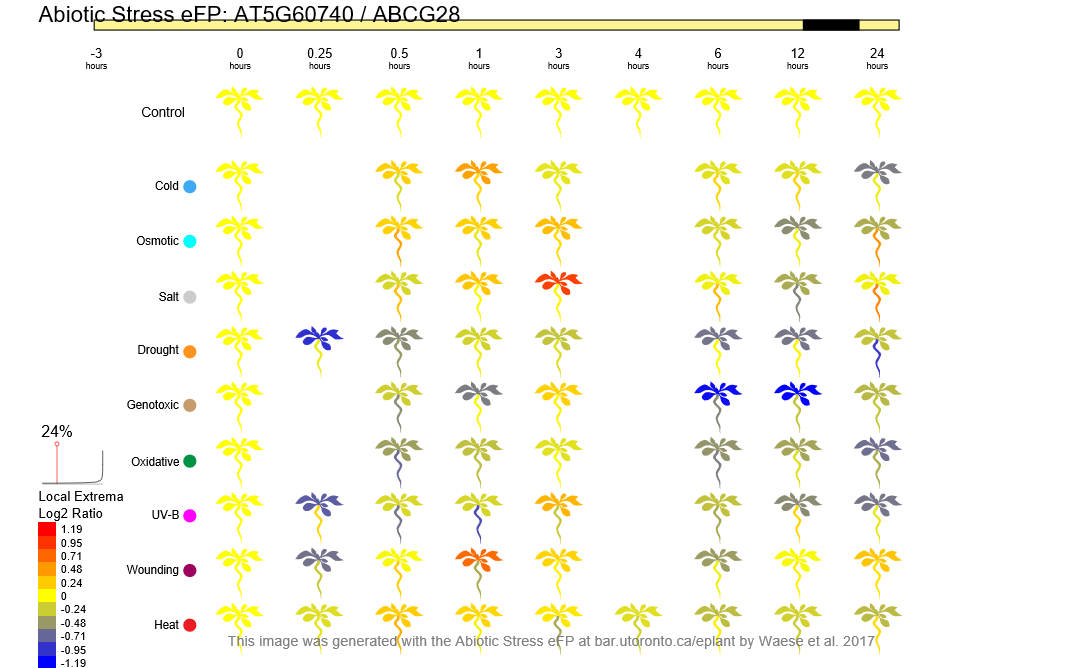


**3A.**


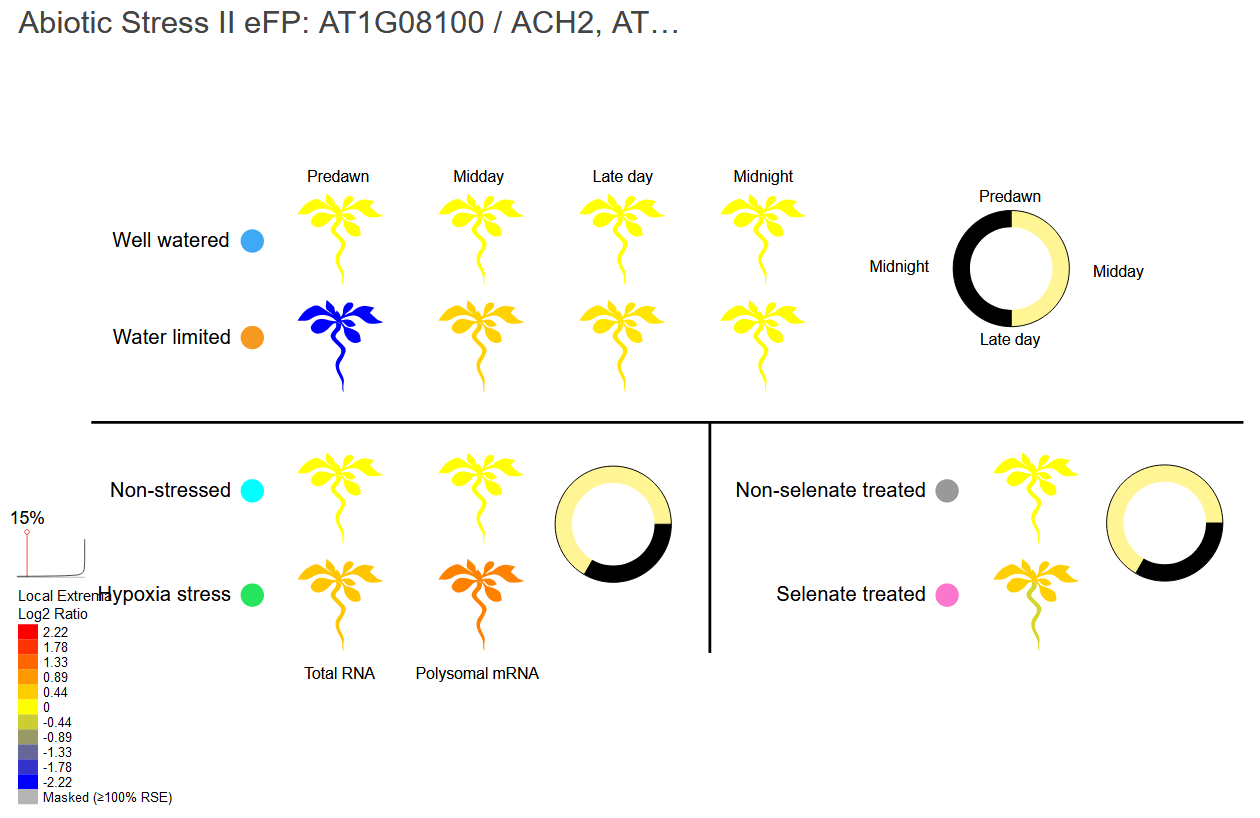


**3B.**
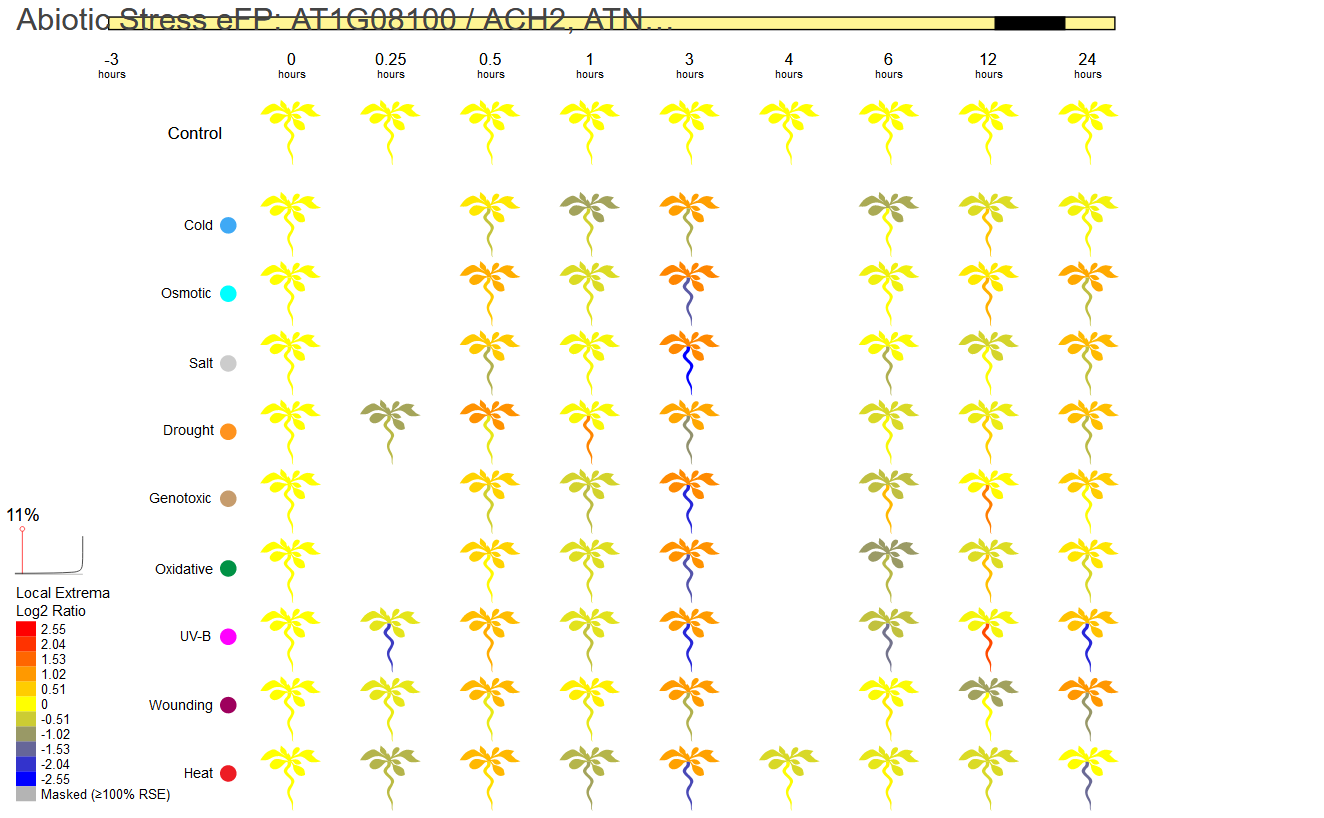


**4A.**

**
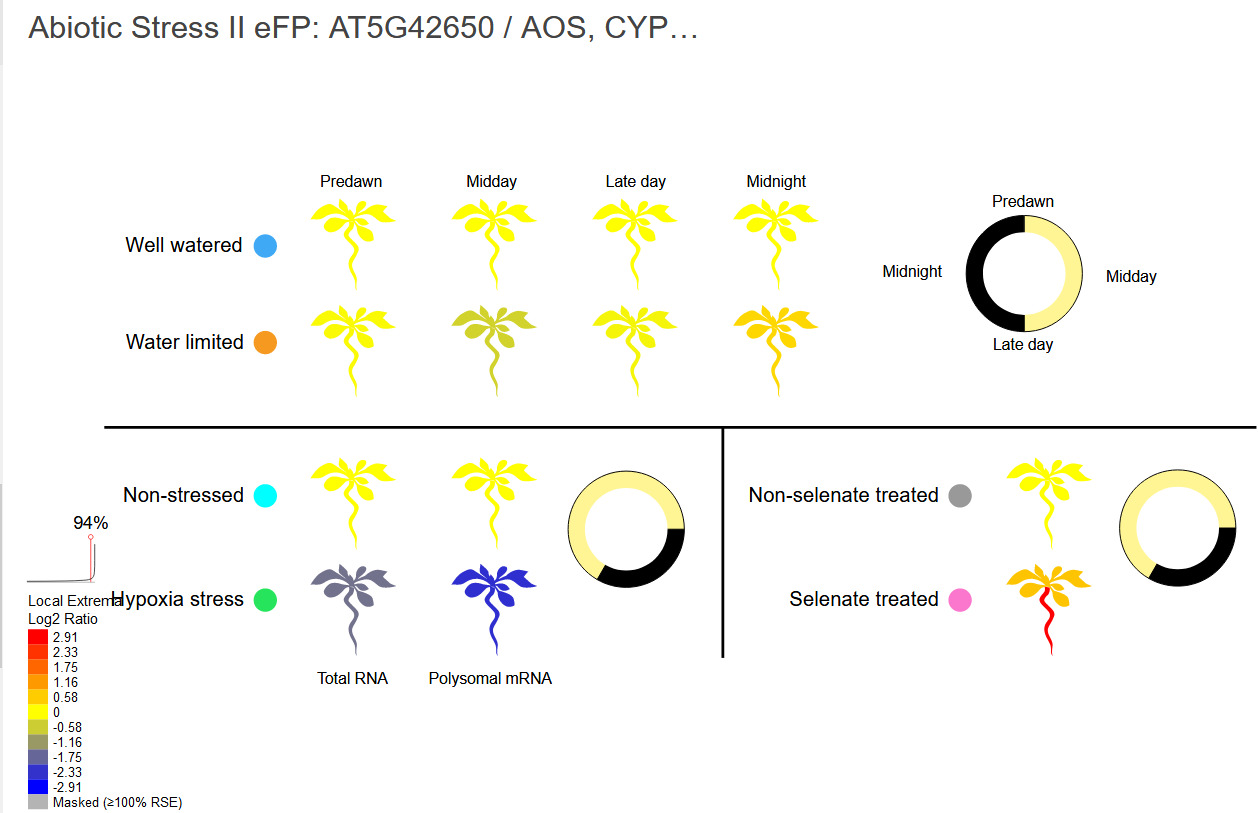
**

**4B.**

**
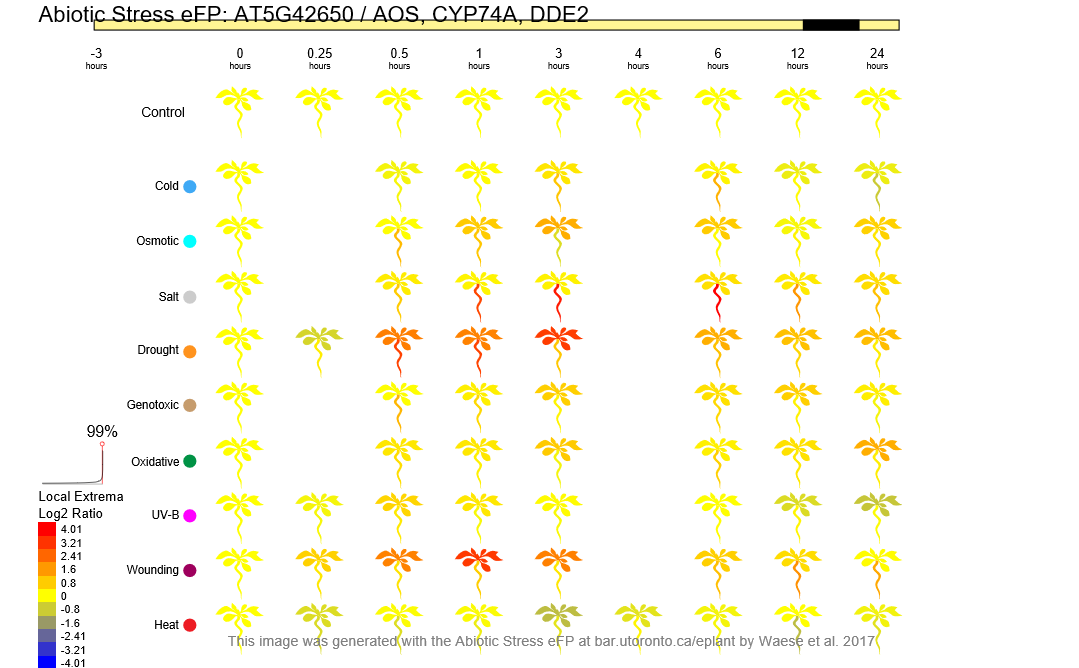
**

**5A.**

**
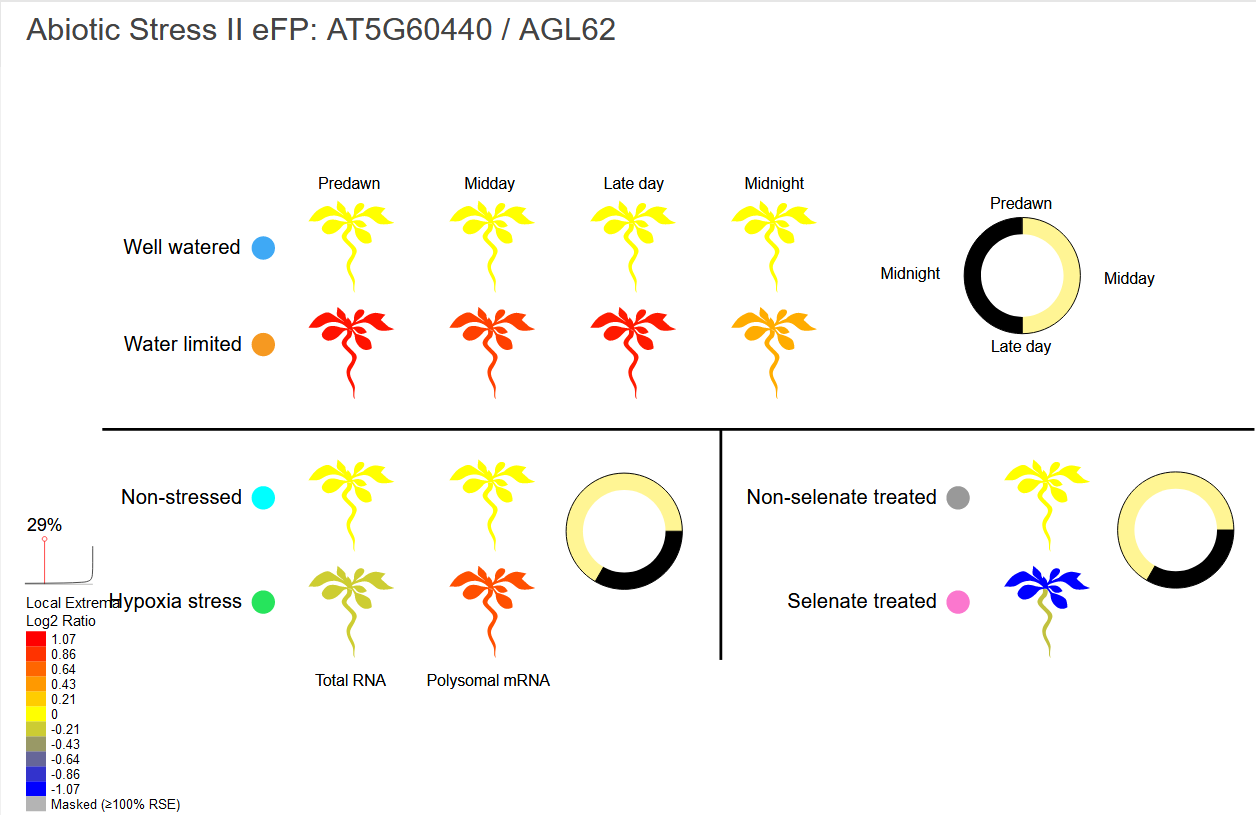
**

**5B.**

**
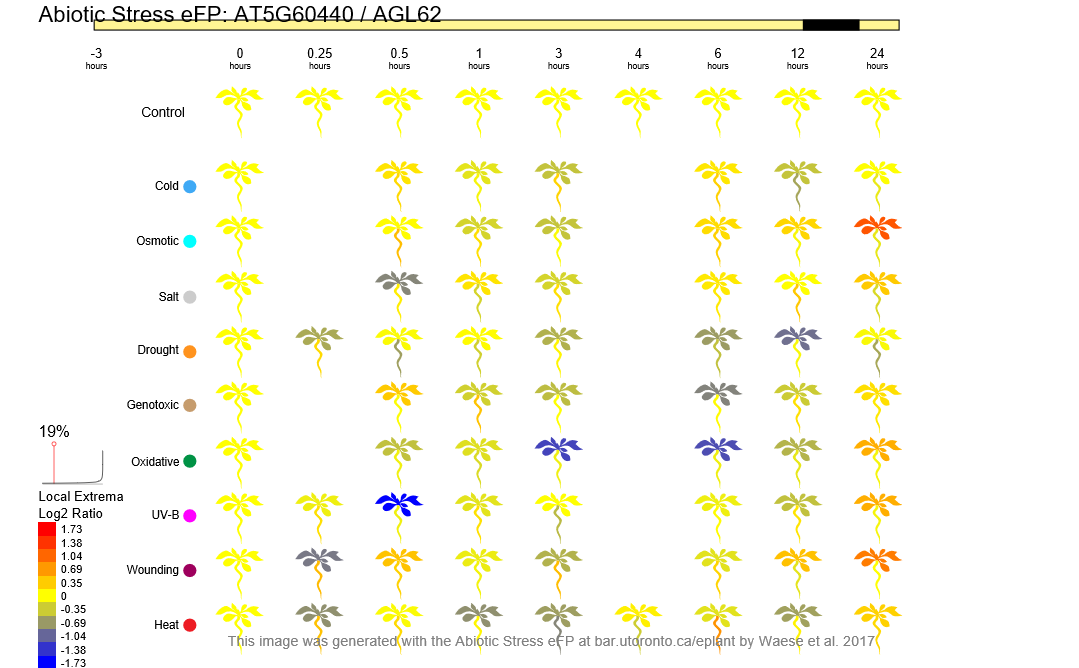
**

**6A.**

**
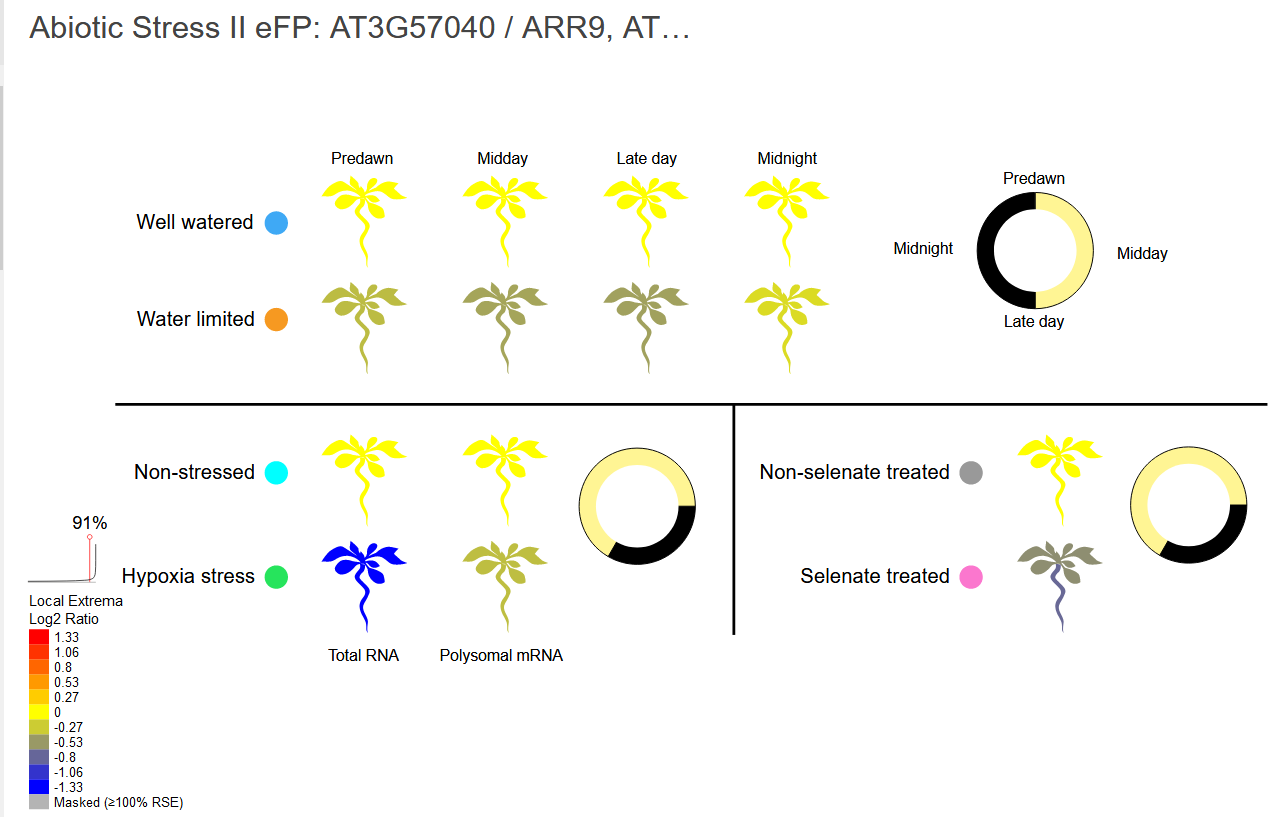
**

**6B.**


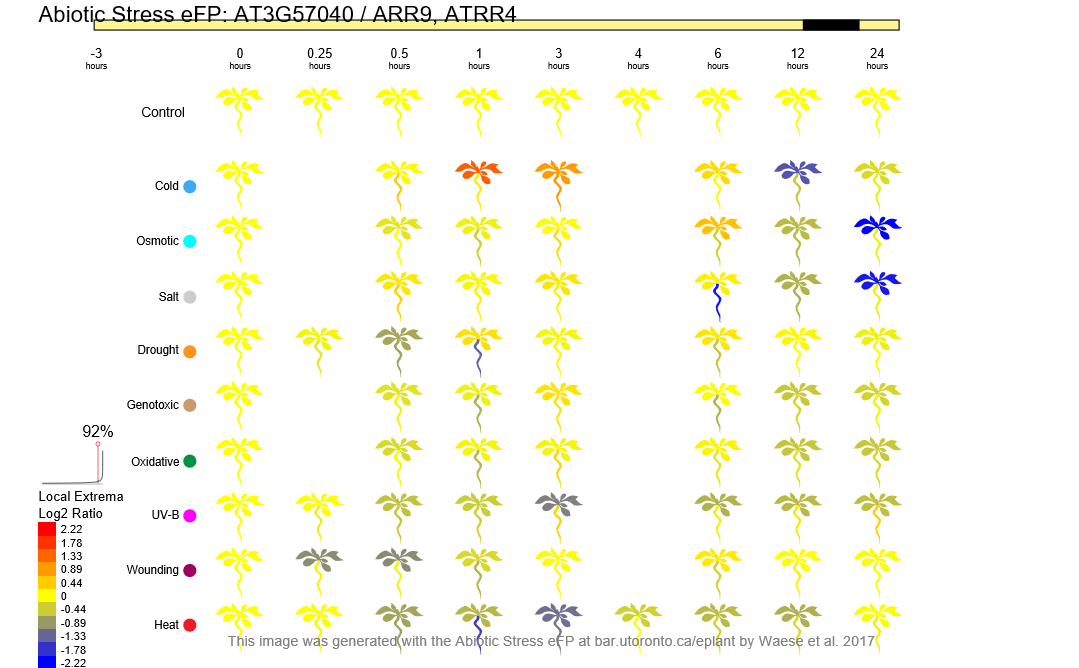


**7A.**

**
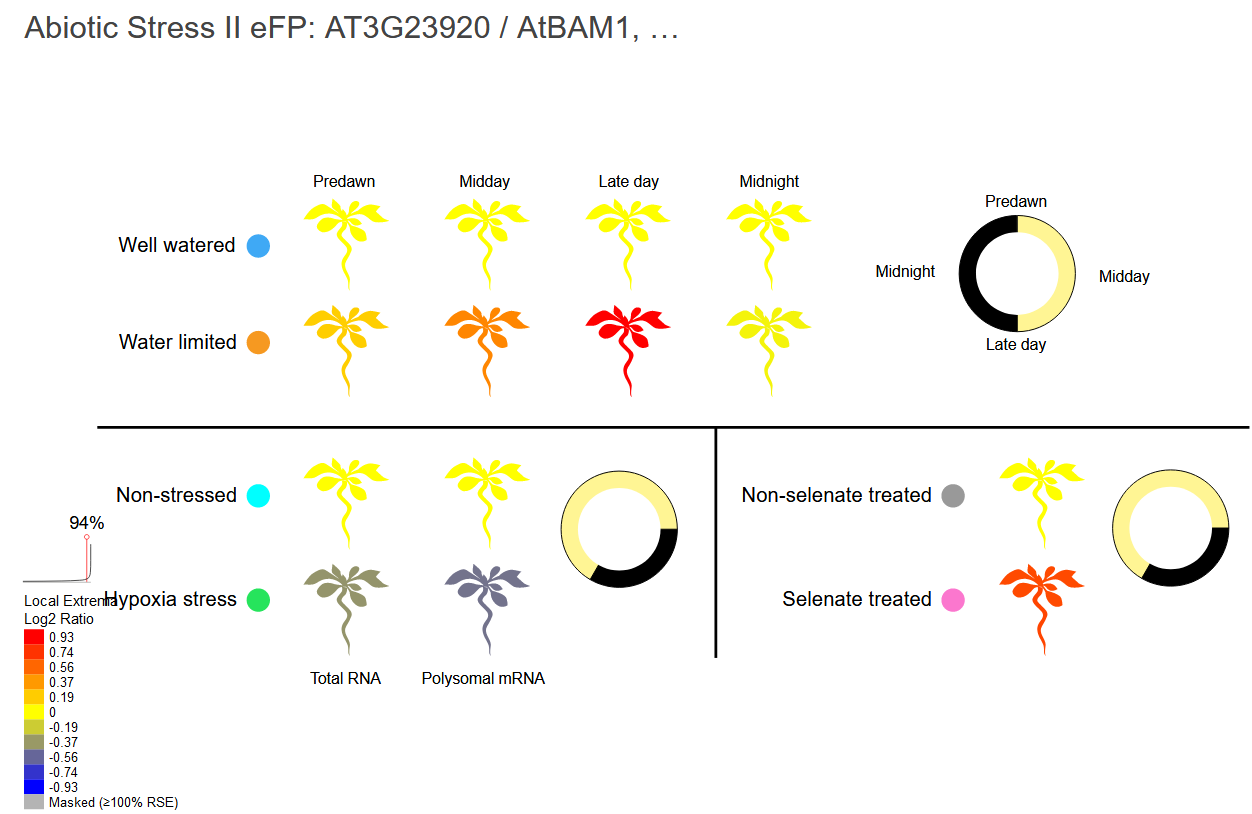
**

**7B.**


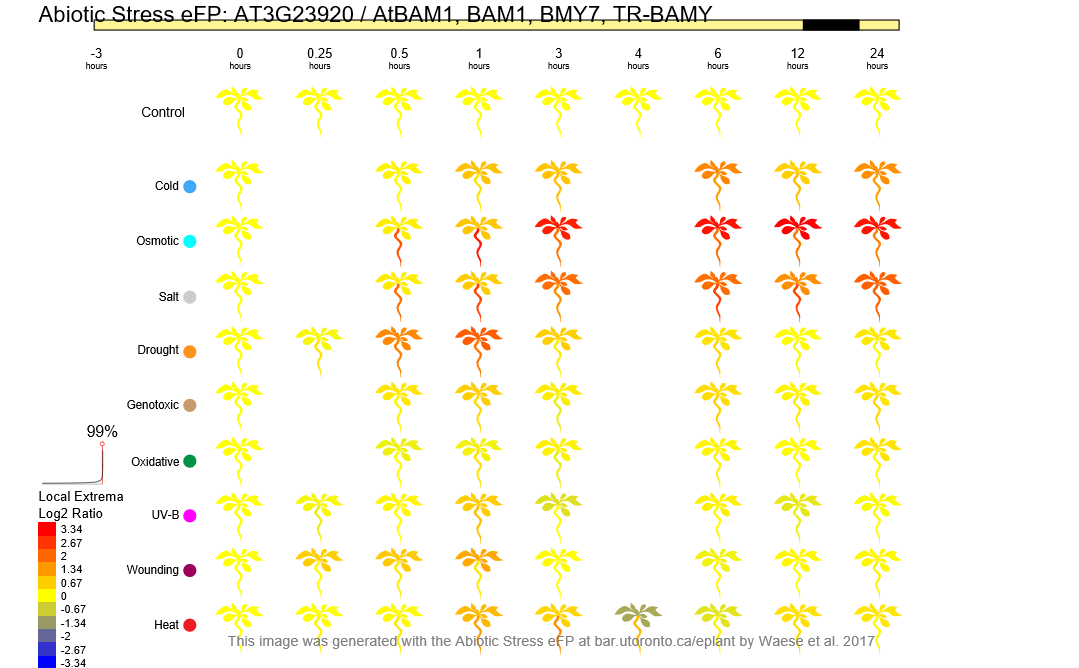


**8A.**


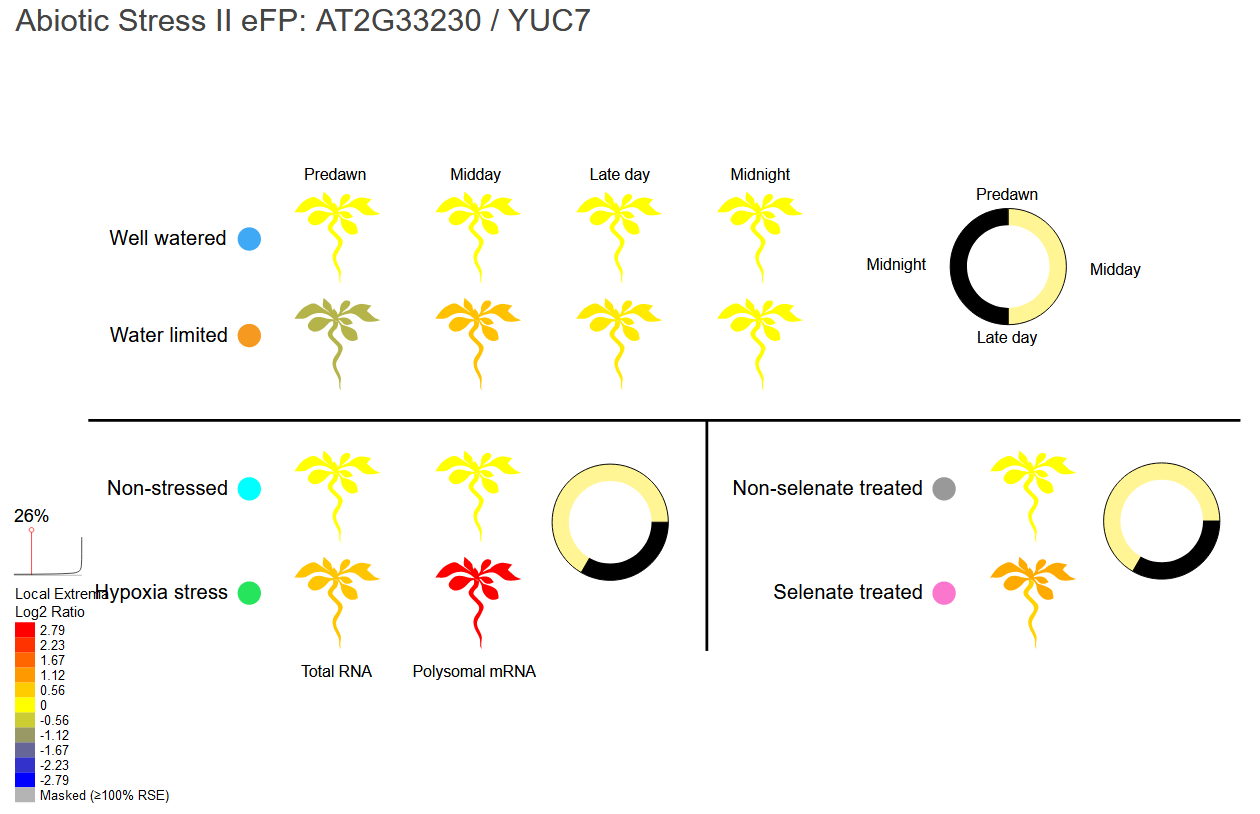


**8B.**


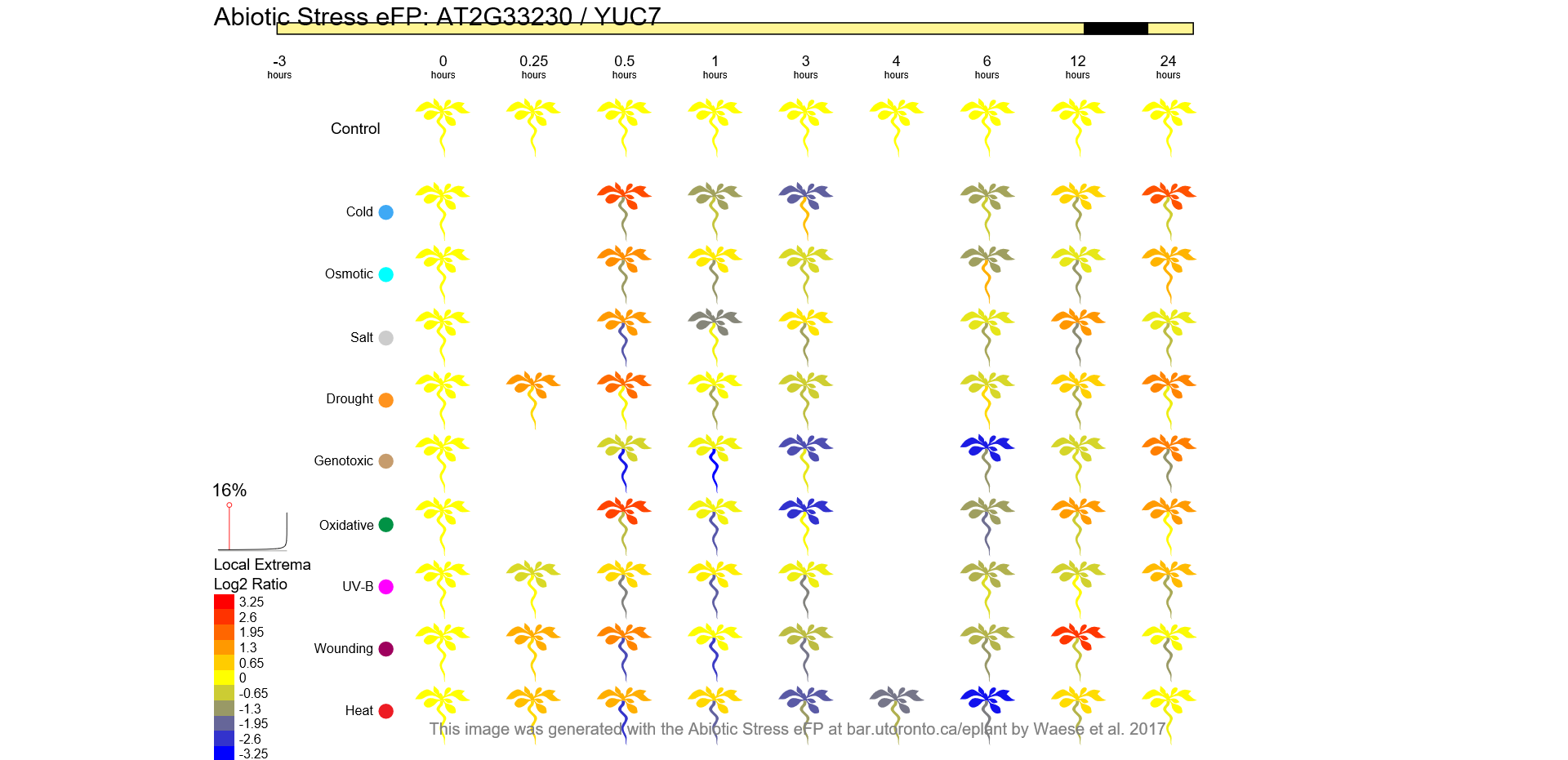


Supplementary Figure SF6: Expression plots obtained from ePlant’s “Abiotic Stress II eFP” (**A**) and “Abiotic Stress eFP” (**B**) views under “Tissue & Experiment eFP viewers” for interesting genes identified in Solanum sitiens inversions against Solanum lycopersicum. Most of these genes are differentially expressed under salt and droughts stresses. **1.** AT1G07960 / Solyc11g069690 / Protein disulfide-isomerase 5-1 **2.** AT5G60740 / Solyc11g069820 / ABC transporter-like **3.** AT1G08100 / Solyc11g069735 / High-affinity nitrate transporter 2.2 **4.** AT5G42650 / Solyc11g069800 / Allene oxide synthase **5.** AT5G60440 / Solyc11g069770 / Agamous-like MADS-box protein AGL62 **6.** AT3G57040 / Solyc10g079600 / Two-component response regulator ARR9 **7.** AT3G23920 / Solyc09g091030 / beta-amylase 1, chloroplastic-like **8.** AT2G33230 / Solyc09g091090 / Probable indole-3-pyruvate.

**References:**

Altschul, S. F., et al. (1990), 'Basic local alignment search tool', *J Mol Biol,* 215 (3), 403-10.

Bartels, Dorothea and Sunkar, Ramanjulu (2005), 'Drought and Salt Tolerance in Plants', *Critical Reviews in Plant Sciences,* 24 (1), 23-58.

Berardini, T. Z., et al. (2015), 'The Arabidopsis information resource: Making and mining the "gold standard" annotated reference plant genome', *Genesis,* 53 (8), 474-85.

Boetzer, M. and Pirovano, W. (2012), 'Toward almost closed genomes with GapFiller', *Genome Biol,* 13 (6), R56.

Boetzer, M., et al. (2011), 'Scaffolding pre-assembled contigs using SSPACE', *Bioinformatics,* 27 (4), 578-9.

Conesa, A. and Gotz, S. (2008), 'Blast2GO: A comprehensive suite for functional analysis in plant genomics', *Int J Plant Genomics,* 2008, 619832.

de Silva, K., et al. (2011), 'Arabidopsis thaliana calcium-dependent lipid-binding protein (AtCLB): a novel repressor of abiotic stress response', *J Exp Bot,* 62 (8), 2679-89.

Ding, S., et al. (2019), 'Identification of Maize CC-Type Glutaredoxins That Are Associated with Response to Drought Stress', *Genes (Basel),* 10 (8).

Dobin, A., et al. (2013), 'STAR: ultrafast universal RNA-seq aligner', *Bioinformatics,* 29 (1), 15-21.

Emms, D. M. and Kelly, S. (2015), 'OrthoFinder: solving fundamental biases in whole genome comparisons dramatically improves orthogroup inference accuracy', *Genome Biol,* 16, 157.

Fernandez-Pozo, N., et al. (2015), 'The Sol Genomics Network (SGN)--from genotype to phenotype to breeding', *Nucleic Acids Res,* 43 (Database issue), D1036-41.

Gao, Y., et al. (2019), 'The Elongation Factor GmEF4 Is Involved in the Response to Drought and Salt Tolerance in Soybean', *Int J Mol Sci,* 20 (12).

Grabherr, M. G., et al. (2011), 'Full-length transcriptome assembly from RNA-Seq data without a reference genome', *Nat Biotechnol,* 29 (7), 644-52.

Gurevich, A., et al. (2013), 'QUAST: quality assessment tool for genome assemblies', *Bioinformatics,* 29 (8), 1072-5.

Han, Xiaojiao, et al. (2017), 'Pathogenesis-related protein PR10 from Salix matsudana Koidz exhibits resistance to salt stress in transgenic Arabidopsis thaliana'.

Jiang, Y. and Deyholos, M. K. (2006), 'Comprehensive transcriptional profiling of NaCl-stressed Arabidopsis roots reveals novel classes of responsive genes', *BMC Plant Biol,* 6, 25.

Jones, P., et al. (2014), 'InterProScan 5: genome-scale protein function classification', *Bioinformatics,* 30 (9), 1236-40.

Kim, D. Y., et al. (2010), 'Overexpression of AtABCG36 improves drought and salt stress resistance in Arabidopsis', *Physiol Plant,* 139 (2), 170-80.

Kleinmanns, J. A., et al. (2017), 'BLISTER Regulates Polycomb-Target Genes, Represses Stress-Regulated Genes and Promotes Stress Responses in Arabidopsis thaliana', *Front Plant Sci,* 8, 1530.

Kumar, M. N., Hsieh, Y. F., and Verslues, P. E. (2015), 'At14a-Like1 participates in membrane-associated mechanisms promoting growth during drought in Arabidopsis thaliana', *Proc Natl Acad Sci U S A,* 112 (33), 10545-50.

Kurtz, S., et al. (2004), 'Versatile and open software for comparing large genomes', *Genome Biol,* 5 (2), R12.

Lee, K. and Kang, H. (2016), 'Emerging Roles of RNA-Binding Proteins in Plant Growth, Development, and Stress Responses', *Mol Cells,* 39 (3), 179-85.

Lee, M., et al. (2012), 'Activation of a flavin monooxygenase gene YUCCA7 enhances drought resistance in Arabidopsis', *Planta,* 235 (5), 923-38.

Li, W. and Godzik, A. (2006), 'Cd-hit: a fast program for clustering and comparing large sets of protein or nucleotide sequences', *Bioinformatics,* 22 (13), 1658-9.

Lovell, J. T., et al. (2015), 'Exploiting Differential Gene Expression and Epistasis to Discover Candidate Genes for Drought-Associated QTLs in Arabidopsis thaliana', *Plant Cell,* 27 (4), 969-83.

Mapleson, D., et al. (2017), 'KAT: a K-mer analysis toolkit to quality control NGS datasets and genome assemblies', *Bioinformatics,* 33 (4), 574-76.

Marcais, G. and Kingsford, C. (2011), 'A fast, lock-free approach for efficient parallel counting of occurrences of k-mers', *Bioinformatics,* 27 (6), 764-70.

Marondedze, C., et al. (2019), 'Changes in the Arabidopsis RNA-binding proteome reveal novel stress response mechanisms', *BMC Plant Biol,* 19 (1), 139.

Osakabe, Y., et al. (2005), 'Leucine-rich repeat receptor-like kinase1 is a key membrane-bound regulator of abscisic acid early signaling in Arabidopsis', *Plant Cell,* 17 (4), 1105-19.

Pakzad, Rambod, et al. (2019), 'Evaluating the antioxidant enzymes activities, lipid peroxidation and proteomic profile changing in UCB-1 pistachio rootstock leaf under drought stress', *Scientia Horticulturae,* 256, 108617.

Patharkar, O. R. and Walker, J. C. (2016), 'Core Mechanisms Regulating Developmentally Timed and Environmentally Triggered Abscission', *Plant Physiol,* 172 (1), 510-20.

Pedranzani, Hilda, et al. (2003), 'Salt tolerant tomato plants show increased levels of jasmonic acid', *Plant Growth Regulation,* 41 (2), 149-58.

Prasch, C. M., et al. (2015), 'ss-amylase1 mutant Arabidopsis plants show improved drought tolerance due to reduced starch breakdown in guard cells', *J Exp Bot,* 66 (19), 6059-67.

Ruan, M. B., et al. (2018), 'Identification and characterization of drought-responsive CC-type glutaredoxins from cassava cultivars reveals their involvement in ABA signalling', *BMC Plant Biol,* 18 (1), 329.

Shelton, J. M., et al. (2015), 'Tools and pipelines for BioNano data: molecule assembly pipeline and FASTA super scaffolding tool', *BMC Genomics,* 16, 734.

Simao, F. A., et al. (2015), 'BUSCO: assessing genome assembly and annotation completeness with single-copy orthologs', *Bioinformatics,* 31 (19), 3210-2.

Smit, AFA., Hubley, R., and Green, P. 'RepeatMasker Open-4.0', <<http://www.repeatmasker.org>>, accessed.

Song, L. and Florea, L. (2015), 'Rcorrector: efficient and accurate error correction for Illumina RNA-seq reads', *Gigascience,* 4, 48.

Stanke, M. and Morgenstern, B. (2005), 'AUGUSTUS: a web server for gene prediction in eukaryotes that allows user-defined constraints', *Nucleic Acids Res,* 33 (Web Server issue), W465-7.

Sweetman, C., et al. (2019), 'AtNDB2 Is the Main External NADH Dehydrogenase in Mitochondria and Is Important for Tolerance to Environmental Stress', *Plant Physiol,* 181 (2), 774-88.

Walker, B. J., et al. (2014), 'Pilon: an integrated tool for comprehensive microbial variant detection and genome assembly improvement', *PLoS One,* 9 (11), e112963.

Wang, Y., et al. (2018), 'Recent advances in auxin research in rice and their implications for crop improvement', *J Exp Bot,* 69 (2), 255-63.

Warren, R. L., et al. (2015), 'LINKS: Scalable, alignment-free scaffolding of draft genomes with long reads', *Gigascience,* 4, 35.

Williams, D., et al. (2013), 'Rapid quantification of sequence repeats to resolve the size, structure and contents of bacterial genomes', *BMC Genomics,* 14, 537.

Wohlbach, D. J., Quirino, B. F., and Sussman, M. R. (2008), 'Analysis of the Arabidopsis histidine kinase ATHK1 reveals a connection between vegetative osmotic stress sensing and seed maturation', *Plant Cell,* 20 (4), 1101-17.

Xie, H., et al. (2016), 'iTRAQ-based quantitative proteomic analysis reveals proteomic changes in leaves of cultivated tobacco (Nicotiana tabacum) in response to drought stress', *Biochem Biophys Res Commun,* 469 (3), 768-75.

Yeo, S., et al. (2018), 'ARCS: scaffolding genome drafts with linked reads', *Bioinformatics,* 34 (5), 725-31.

Yu, G., et al. (2018), 'Two Methods for Mapping and Visualizing Associated Data on Phylogeny Using Ggtree', *Mol Biol Evol,* 35 (12), 3041-43.

Zanella, M., et al. (2016), 'beta-amylase 1 (BAM1) degrades transitory starch to sustain proline biosynthesis during drought stress', *J Exp Bot,* 67 (6), 1819-26.

Zdobnov, E. M., et al. (2017), 'OrthoDB v9.1: cataloging evolutionary and functional annotations for animal, fungal, plant, archaeal, bacterial and viral orthologs', *Nucleic Acids Res,* 45 (D1), D744-D49.

Zhang, H., et al. (2017), 'Transcript profile analysis reveals important roles of jasmonic acid signalling pathway in the response of sweet potato to salt stress', *Sci Rep,* 7, 40819.

Zhao, J., et al. (2013), 'A receptor-like kinase gene (GbRLK) from Gossypium barbadense enhances salinity and drought-stress tolerance in Arabidopsis', *BMC Plant Biol,* 13, 110.

Zhong, L., et al. (2015), 'AtTGA4, a bZIP transcription factor, confers drought resistance by enhancing nitrate transport and assimilation in Arabidopsis thaliana', *Biochem Biophys Res Commun,* 457 (3), 433-9.

Zhu, C., et al. (2014), 'Molecular characterization and expression profiling of the protein disulfide isomerase gene family in Brachypodium distachyon L', *PLoS One,* 9 (4), e94704.

Zimin, A. V., et al. (2013), 'The MaSuRCA genome assembler', *Bioinformatics,* 29 (21), 2669-77.
